# Mindreading quality versus quantity: A theoretically and empirically motivated two-factor structure for individual differences in adults’ mindreading

**DOI:** 10.1371/journal.pone.0305270

**Published:** 2024-06-25

**Authors:** Christina Pomareda, Rory T. Devine, Ian A. Apperly

**Affiliations:** School of Psychology, University of Birmingham, England, United Kingdom; Bangladesh Open University, BANGLADESH

## Abstract

Existing methods for studying individual differences in adults’ mindreading often lack good psychometric characteristics. Moreover, it remains unclear, even in theory, how mindreading varies in adults who already possess an understanding of mental states. In this pre-registered study, it was hypothesised that adults vary in their motivation for mindreading and in the degree to which their answers on mindreading tasks are appropriate (context-sensitive). These factors are confounded in existing measures as they do not differentiate between the frequency of mental state terms (MST), indicative of motivation, and the quality of an explanation. Using an innovative scoring system, the current study examined whether individual differences in adult undergraduate psychology students’ (*N* = 128) answer quality and / or quantity of explicit references to others’ mental states on two open-ended response mindreading tasks were separable constructs, accounted for by mindreading motivation, and related differentially to measures previously linked with mindreading (e.g., religiosity, loneliness, social network size). A two-factor and one-factor model both provided acceptable fit. Neither model showed significant associations with mindreading motivation. However, a two-factor model (with MST and response appropriateness loading onto separate factors) provided greater explanatory power. Specifically, MST was positively associated with religiosity and response appropriateness was negatively associated with religiosity, whilst the one-factor solution did not predict any socially relevant outcomes. This provides some indication that mindreading quantity and mindreading quality may be distinguishable constructs in the structure of individual differences in mindreading.

## Introduction

Mindreading is a concept central to social cognition. Individual differences in the ability to attribute mental states such as thoughts, beliefs, or desires to others correctly and to explain and predict behaviour taking these mental states into account, have been linked with many positive outcomes such as lowered aggression [1] and reduced stereotyping [2]. However, it is still unclear how and why mindreading varies in adults who already possess core mental state concepts of their own and others’ thoughts, beliefs, and feelings [3]. In addition, more recent research has emphasised that motivational factors impact the manifestation of this capacity and its resulting benefits [4, 5]. The present study was based on the consideration that adults might vary independently in their motivation for mindreading and in the degree to which their answers on mindreading tasks are appropriate. These factors have been proposed to be conceptually and empirically separate [4–6] but are confounded in existing open-ended response measures of mindreading, which typically do not differentiate between the *quantity* of mental state terms (MST) and the *quality* of explanations given. The quantity of references to thoughts, feelings and other mental states is a potential indicator of motivation, whereas the *quality* of explanations indicates the contextual appropriateness of the inferences. Quantity and quality are clearly dissociable in principle because individuals may mentalize about someone else but do so in a way that seems at odds with a given situation. Testing whether they are distinguishable in practice addresses a current gap in the literature and can advance theory by casting light on the underlying sources of variation in adults’ mindreading, as well as how they might be measured. The overall aim of the present study was to examine whether *individual differences* in adults’ response quality and / or quantity of MST on two open-ended response mindreading tasks were (a) separable constructs, (b) accounted for by differences in individuals’ mindreading motivation, and (c) related differentially to a variety of outcome measures that had previously been empirically or theoretically linked with mindreading; religiosity, authoritarianism, loneliness, social network size and anthropomorphism [7–9].

### Measuring individual differences in adults’ mindreading

Developmental research into mindreading has been dominated by questions about *when* and *how* children acquire mindreading concepts. Given that older children and adults already have a conceptual understanding of what thoughts, beliefs and desires are, that they may differ between people, and that they can impact behaviour, researchers have devised a wide range of tasks that are more subtle and complex in nature to assess mindreading in middle childhood and adolescence [10] and in adulthood [11]. However, mindreading tasks devised to be complex enough to capture individual differences in adults’ mindreading are often characterised by a lack of convergent validity, i.e., performance on one measure of mindreading does not correlate with performance on another measure [12, 13] or construct validity (i.e., the same task can be used to measure different constructs) [14, 15]. Some tasks used to assess mindreading such as the Animations Task [11, 16], which requires participants to explain the movements of triangles that exhibit either a simple interaction (e.g., two triangles appearing to be dancing), or a more complex interaction (e.g., one triangle appearing to surprise the other triangle), are *sometimes* also utilised to measure other constructs, such as anthropomorphism [17, 18]. Similarly, the Reading the Mind in the Eyes test (RMET) [19], originally developed to measure mindreading, may be more suitable as a measure of emotion perception given that the task requires participants to match suitable adjectives with pictures of faces expressing emotions [14, 15]. It is therefore unclear what “advanced” mindreading tasks really measure [14, 15, 20, 21]. Furthermore, mindreading research in adults has predominantly focused on group-level comparisons between clinical and non-clinical groups [12, 22, 23] and does not provide satisfactory answers as to whether and why there are *individual differences* in adults’ mindreading.

Notwithstanding these limitations, some mindreading tasks have also been successfully administered to typical adults [12, 24–26]. The Movie for the Assessment of Social Cognition [MASC, 26] requires participants to reason about the mental states of four characters who are shown in a realistic social interaction based on a multiple-choice question regarding the characters’ mental states. Neurotypical adults have been found to select correct responses approximately 60% of the time [23, 26], which indicates that the MASC is suitable for measuring individual differences in non-clinical adult samples. It should, however, be noted that the multiple-choice format of the MASC does not allow for the differentiation of frequency of mental state terms; a quantitative marker of an individual’s tendency to mindread based on the frequency with which they refer to other people’s mental states, and the appropriateness of participants’ mindreading responses; contextually justified mindreading.

The Silent Films Task [SFT; 10] has specifically been developed to detect individual differences in mindreading beyond the pre-school years. The task requires participants to answer six open-ended questions about five video clips that portray a main character interacting with a range of peripheral characters. Although this task exhibits convergent validity (e.g., scores on the SFT are strongly correlated to scores on another–strange stories–task) [10], criterion validity [27], and test-retest reliability [28], it has yet to be formally evaluated beyond early adolescence. Furthermore, the original questions used in the SFT prompt participants to reason about mental states and do not differentiate between frequency of MST and response appropriateness. Thus, despite these advances in the creation of “advanced” mindreading tasks, challenges persist in the measurement of how mindreading *varies* in adults [3, 12, 14, 15, 20, 29].

Finally, there are grounds to think that frequency of MST and response appropriateness may be separable constructs, although they are often confounded in open-ended response coding schemes. The frequency of spontaneously produced MST is distinct from performance on mindreading tasks in middle childhood [30], in adults [24], and in older adults [31]. Furthermore, there is evidence that the use of mental state terms can vary with different interaction partners [31], perhaps because some social interactions are more motivating than others.

### The role of motivation in individual differences in adults’ mindreading

A person’s motivation fundamentally shapes their behaviour [32]. Indeed, making mentalistic attributions is an effortful process that places high demands on memory and cognitive control [33–36]. Individual differences in motivation might therefore also influence performance on mindreading tasks [4, 5, 6, 32].

Mindreading motivation has been defined as a stable orientation to engage effortfully with others’ minds and mental states [4], and it has been suggested that autistic people sometimes perform less well on mindreading tasks because they have lower social motivation rather than lower social ability [37–39]. Likewise, there is evidence that neurotypical adults’ mindreading motivation is positively related to their performance on mindreading tasks [4]. Specifically, Carpenter et al. (2016) found small positive correlations between mindreading motivation, assessed via the 13-item Mind Reading Motivation Scale and performance on the interpersonal perception test 15 [IPT-15; 4], which requires participants to make judgements videotaped real-world social situation. It has been suggested that mindreading motivation and mindreading ability are conceptually and empirically separable [4–6], and there is evidence that social motivation and mindreading ability make distinctive contributions to explaining social outcomes [5]. The present study combined this observation with a revised coding scheme for mindreading that distinguishes between the quantity of mental state terms and the appropriateness of mindreading responses.

Overall, the above-described concerns and previous research findings motivate the first objective of introducing a scoring system that distinguishes between (a) mindreading quantity, a potential indicator of motivation, operationalised as frequency of MST in responses, and (b) mindreading quality, operationalised as the “appropriateness” of participants’ answers when responding to open-ended versions of the Silent Films Task [SFT; 10] and the Movie of the Assessment of Social Cognition (MASC; 26).

### Criterion validity of MST and response appropriateness

One way to distinguish between two closely related constructs is to investigate differential relations between the constructs and other variables [4]. Although previous results have not always been consistent and may have been subject to some of the psychometric shortcomings of mindreading tasks [12, 21], they provide potential criteria for evaluating the different factor structures used to conceptualise mindreading in the present study. The distinctiveness of individual differences in mindreading quality and mindreading quantity was therefore examined by investigating whether these constructs might show unique associations with variables that have been linked with mindreading performance, such as measures of *social competence* [10, 7, 40] and *personality tendencies* such as religiosity [41–43], authoritarianism [44], and anthropomorphism [17, 45].

### The present study

In summary, we sought to shed light on the nature of individual differences in mindreading in adults, by creating a novel scoring system that leveraged existing open-ended mindreading tasks. To this end, whether mindreading quantity can be distinguished from mindreading quality was assessed. More specifically, the present study examined whether individual differences in neuro-typical adults’ (*N* = 128) and frequencies of explicit references to others’ mental states and response appropriateness on two open-ended response mindreading tasks were (a) separable constructs, (b) accounted for by differences in individuals’ mindreading motivation, and (c) related differentially to a variety of outcome measures that had previously been empirically or theoretically linked with mindreading.

It was hypothesised that if frequencies of MST and response appropriateness are separable constructs, then response appropriateness and frequencies of MST will load onto separate latent factors (i.e., one representing response appropriateness and one frequencies of MST, a potential indicator of motivation). Alternatively, if frequencies of MST and response appropriateness test the same underlying construct (i.e., “mindreading”), response appropriateness and frequencies of MST will load onto one latent factor. Finally, it is possible that individual differences in performance will be driven by characteristics of the two different tasks, rather than the psychological constructs of response appropriateness, frequencies of MST or mindreading. This effect would lead indicators (i.e., MST and appropriateness) coded from the SFT to load onto one latent factor (i.e., representing performance on the SFT) and both indicators coded from the MASC to load onto a separate factor. The possibility of shared method variance will be accounted for in our analyses. Furthermore, it was hypothesised that mindreading motivation will be specifically related to the quantity of mental state terms produced in participants mindreading responses.

## Methods

Sample size calculations, all data exclusions, all manipulations, and all measures that were used in the study are reported.

### Participants

This study was pre-registered prior to the start of data collection. This is a link to the preregistration: https://osf.io/uerkx/). A total of 133 participants were recruited between the 24^th^ of January and 28^th^ of March 2021 (the link to the study was accessible between these dates) via word of mouth and via the University of Birmingham School of Psychology undergraduate database.

Power-analysis based on an a-priori sample size calculator for structural equation models [46] indicated that a minimum sample size of 119 participants was needed to obtain a level of acceptable statistical power of .80, and for a model that has a maximum of three latent variables and 15 indicator and observed variables. Our minimum target sample size was therefore 119 participants. All participants took part in the study in exchange for course credit and were psychology students. To be eligible participants had to be at least 18 years old, report no current or past neurological or psychiatric problems, and be English-speaking (including native and non-native English speakers). Based on these pre-registered exclusion criteria, 5 participants were excluded due to reporting a past neurological or psychiatric problem, leaving a final sample of 128 participants (113 females, 15 males, *M*age = 19.47 years, *SD* = 1.47 years, range = 18–28). The participation rate was 96.24%. One hundred and seven participants (83.6%) reported English as their native language, with 71.9% of participants (*N* = 92) being monolingual, 25% (*N* = 32) bilingual and 3.1% (*N* = 4) multilingual (speaking more than two languages). Most participants (95.3%, *N* = 122) had completed secondary school or equivalent (e.g., A levels), 2.3% (*N* = 3) held a bachelor’s degree, and 1.6% a master’s degree (*N* = 2). Participants were invited to identify their ethnicity according to the descriptions recommended by the United Kingdom Office for National Statistics (ONS) that are comprised of five broad categories and 18 sub-categories. In order of size, the five broad categories were presented as follows: 70.4% (*N* = 90) of participants identified as White, 16.5% (*N* = 21) identified as Asian, 5.5% (*N* = 7) as Black, 4.7% (*N* = 6) as Mixed, and one participant (0.8%) as an ethnicity not listed. Data on religion was also recorded according to the questions proposed by the ONS: 56.3% (*N* = 72) of participants reported no religion, 21.1% (*N* = 27) identified as Christian, 2.3% (*N* = 3) of participants as Hindu, 7% (*N* = 9) as Jewish, 10.2% (*N* = 13) as Muslim, 1.6% (*N* = 2) as Sikh, and 1.6% (*N* = 2) preferred not to answer the question.

### Design and procedure

As this was an individual differences study, questionnaires and measures were administered in a fixed order, following Goodhew and Edwards (2019) [47]. The rationale is that a fixed order of presentation makes it more likely that any effects of task order are equivalent for all participants, which minimizes the unexplained variance when the focus of analysis is on the pattern of individual differences between participants. A fixed order of presentation of course raises the possibility that fatigue or practice, or order effects, will result in unreliable measurement. For example, highly fatigued participants might show floor-level performance, or answer at random. All measures were therefore checked for reliability. All questions were administered via the Qualtrics survey platform. Testing was completed in one session and no feedback was provided in relation to response content. The time participants had to complete the tasks was not limited and the total duration of the study was approximately one hour. Informed written consent was obtained from all participants prior to participating and the study was approved by the Ethical Review Committee at the (*Masked*) (protocol code: ERN_09–719 2020). The recruitment system and software for online testing ensured that participants’ identities were never known to the researchers. Participants’ data was stored confidentially and only available to the researchers involved in this study at the University of Birmingham.

## Materials

### Response appropriateness and frequencies of Mental State Terms (MST)

#### Movie Assessment of Social Cognition [MASC; 26]

Participants watched a 15-minute movie, which was paused several times to ask participants both the open-ended (e.g., *“What do you think happened during this clip*?*”*) as well as the original multiple-choice question following the open-ended question. The number of questions was reduced from the original 45 (assessed via multiple-choice questions) to 10. Participants watched the whole movie, but the movie was paused only 10 times, with the remaining clips merged, to account for the fact that the questions were open-ended.

Multiple-choice answers were scored as either correct or incorrect, with further differentiation of the incorrect answers into over-mentalizing (overly complex mental-state reasoning, e.g., ‘she is exasperated about Michael coming on too strong’), no mentalising (failing to make a mental state attribution) and under-mentalising (overly simplistic mental state inferences, e.g., ‘she is pleased about his compliment’). Scores based on the multiple-choice questions were retained for comparison purposes.

To rate response appropriateness, a novel coding scheme for participants’ answers to open-response questions was developed. Two points were awarded for each one of the ten clips, if two pre-specified criteria deemed to capture the essence of the actions of the clip were met. Explicit references to mental-state terms were not required to achieve full scores, however, participants were required to either directly or indirectly attribute mental states to a character in the clips in a way that was consistent with the events shown and reflective of the question asked. Appropriate responses had to include a reference to both interacting entities. Furthermore, responses could not include speculation that could not have been logically derived from the clip. One point (reflecting a partially appropriate response) was awarded if a response met one of the two criteria. To achieve one point, participants’ description had to still be related to the sequence and show rudimentary understanding of the reasons for the characters’ actions without, however, reaching full understanding (i.e., responses may have been imprecise or incomplete). Zero points were awarded if none of the two criteria were met. In general, such responses were reflective of participants misunderstanding the question, were nonsensical, incoherent and / or focussed on minor aspects of the sequence. Zero-point responses would not enable the reader to reconstruct the sequences of the video clip.

Our coding approach draws from Castelli et al. (2001) [11] who coded participants’ descriptions of animated triangle cartoons in terms of coherent retelling of the scene (which they called “appropriateness”) and a separate score for mindreading (which they called “intentionality”). Our approach is distinctive because it distinguishes between the appropriateness and quantity of mindreading. An “appropriate” response required evidence of contextually justified mindreading, whereas MST quantified the number of mental state terms regardless of their appropriateness. Across all 10 items of the MASC participants could score from 0 (reflecting exclusively inappropriate responses) to 20 (reflecting exclusively appropriate responses). Inter-item reliability for the appropriateness ratings of the MASC ranged from ICCs of .87 to .92. The number of references participants made to the characters’ mental states was also recorded. Following the approach taken by previous studies [48], the coding scheme for MST used in the present study was adapted from existing coding schemes [11] and extended with words that fit the general definition of what constitutes a mental state term but were not explicitly named in the existing coding schemes considered. Specifically, participants’ references to their own mental states (e.g., “I think”) were not record. The total number of words within each response was also counted. All verbs, nouns or adjectives referring to cognitions such as “think”, “remember”, “know”, desires such as “want” or “like” (not as a preposition) or emotion such as “happy”, “excited”, “angry” were coded as MST. MST was not utilised as a proportion of response length variables in the present study but the raw scores were used instead. Inter-rater reliability was calculated for 20% of items (*N* = 25) and was acceptable, with ICCs ranging from .79 to .99. The coding manual for the MASC is available on the OSF (https://osf.io/uerkx/).

**Silent Film Task [10].** Participants watched five short black and white silent film clips showing one main character interacting with several different peripheral characters. Across the five clips, participants were asked an open-ended question (i.e., *“What do you think happened during this clip*?”) after each clip. This was followed by a clip-specific question which had originally been developed for administering the task to children and adolescents (e.g., *“Why do you think the men hide*?*”)*. For the original questions, responses were coded using the standard rating scheme and retained for comparison purposes only as part of the same, within-participant, study. The measure and standard scoring manual are available at the OSF: https://osf.io/8x73r/.

Our critical data came from an open-ended question after each clip. In line with how response appropriateness was coded for the MASC in the present study, participants’ responses to this question were coded using a novel 3-point appropriateness scale indicating appropriate, partially appropriate, or inappropriate understanding. Points for the open-ended question were awarded according to the same logic as for the MASC (see description above). Specifically, for this item, to achieve two points, participants needed to (a) describe both Harold’s (sits in the van) and the driver’s (drives away) behaviour and (b) recognise that the driver did not know (e.g., pay attention, realise) that Harold was in the van. This could have been implicit via stressing that the driver was deaf in relation to him driving away. One point was awarded for each element, and zero points were awarded if neither element was mentioned. Across all items, minimally, participants could score 0 points and, maximally, 10 points could be achieved. Summed scores were normally distributed. Items showed acceptable inter-rater reliability, *N* = 25, with ICCs ranging from .85 (for item three) to .93 (for item four). In addition, the number of mental state terms contained for each item of the SFT was counted. Inter-item reliability was acceptable, *N* = 25, with ICCs ranging from .74 (for item two) to .97 (for item three). The coding manual for the SFT can be found on the OSF (https://osf.io/uerkx/?view_only=a291a3440d814c3c8407fe94ad78f83b).

#### Mindreading Motivation (MRM) scale [4]

Participants rated their level of agreement on a scale ranging from 1 (disagree completely) to 7 (agree completely) for 13 questions assessing the degree to which they perceive themselves as oriented towards effortfully engaging with others’ perspectives and mental states. As per the standard scoring scheme, items 2, 4, 5, 7, 9, 10, 11, 12, and 13 were reversely coded. Across all items, participants could minimally score 13 points and maximally 9, α = .67.

#### Religiosity

Based on the United Kingdom Office for National Statistics proposed questions, participants indicated their religious affiliation, based on the question *“What is your present religion*, *if any*?*”*. No religion was coded as 0 and having a religious affiliation was coded as 1. Religious Practice was assessed via the question *“Aside from weddings and funerals*, *how often do you attend religious services*?*”*, with response options ranging from 1 (More than once a week) to 6 (Never). To assess religious upbringing, participants were asked *“As a child*, *were you raised in a religious home*?*”*, with response options of “Yes”, “No” and “Don’t know”. Religious belief was assessed with the following question: *“Which statement comes closest to expressing what you believe about God*?*”*. Across all items, higher scores indicated greater religiosity, α = .68.

#### Social networks [7]

To measure individual differences in *social network size*, participants were asked to list the initials of everyone they were in social contact within the last month, either over the telephone, text message, video communication software, email or in person. Participants were asked to exclude purely work-related contacts or casual acquaintances such as someone they have briefly encountered in the street. The total number of contacts listed was summed (*M* = 18.64, *SD* = 12.03, *min* = 1, *max* = 110). *Advice network size* (i.e., the number of people a participant discussed important matters with) was also summed, *M* = 8, *SD* = 1.77, *min* = 2, *max* = 15. Participants were able to write up to ten people’s initials and were further instructed to indicate within these contacts how emotionally close they felt to their advice network contacts on a scale ranging from 1 (not close at all) to 5 (extremely close), with higher scores indicating greater closeness. The resulting scores were summed to create an overall emotional closeness score for each participant. This variable was normally distributed, *M* = 29.52, *SD* = 7.67, *min* = 10, *max* = 50.

#### Individual Differences in Anthropomorphism Questionnaire [IDAQ; 8]

Across 15 items, participants rated the extent to which natural entities, non-human animals and technological devices have “free will”, “consciousness”, “a mind of its own”, “intentions” and can “experience emotions”. Responses are rated on a scale from 0 (not at all) to 10 (very much). IDAQ (α = .85) scores were created according to the standard coding for each participant. Scores for the IDAQ scale ranged from 18 to 154. The data was normally distributed.

#### Revised UCLA Loneliness Scale (RULS-6) [9]

Participants were asked to respond on a 4-point Likert scale ranging from 1 = “often” to 4 = “never” to six questions, for example, *“How often do you feel that you lack companionship*?*”*, assessing the degree to which participants felt lonely, α = .897. The degree of loneliness was evaluated by averaging scores across items. The items were reverse-coded, with higher scores indicating greater loneliness. Loneliness average scores ranged from 1 to 4 and the data was normally distributed, α = .67.

### Ten Item Personality Measure (TIPI) [49]

The 10-item measure of the Big Five (or Five-Factor Model) dimensions of personality was administered. Participants indicated the degree to which they ascribed different personality traits to themselves, using a 7-point Likert scale ranging from 1(Disagree strongly) to 7(Agree strongly). Items 2, 4, 6, 8 and 10 were reversely coded (e.g., for these items 7 was recoded with a 1, a 5 with a 3 etc.). The average of two items (standard item and reverse-scored item) made up a scale (e.g., extraverted, enthusiastic & reserved, quiet, a = .77, Critical, quarrelsome & Sympathetic, warm, a = .29, Dependable, self-disciplined & Disorganised, careless, a = .64, Anxious, easily upset & Calm, emotionally stable, a = .65, Open to new experiences, complex & Conventional, uncreative, a = .37).

### Wechsler abbreviated scale of intelligence [50]

Current intellectual functioning (verbal ability) was assessed using the Similarities Test of the Wechsler Adult Scale of Intelligence. This vocabulary subtest has been recognized as among the most widely used measure of general mental ability. Participants completed 20 trials (with 20 items), explaining why two words were alike. For example, participants were asked: “In what way are grapes and strawberries alone?”, to which the correct answer would be “both fruit”. Responses were scored using the WASI manual and items were scored on a 3-point scale from 0 to 2. Scores were added up, with higher scores indicating greater expressive language ability, α = .33, *range* = 22–35.

## Results

### Analysis plan

First, measurement models for mindreading appropriateness (i.e., response appropriateness for the MASC and SFT) and frequencies of mental state terms (from responses to the MASC and SFT) were tested. Following this, whether mindreading motivation as well as a range of outcome measures were associated with individual differences in mindreading using structural equation modelling was examined. The latent factors were regressed onto age, gender (0 = male, 1 = female), education, whether participants spoke English as their native language (0 = no, 1 = yes) and verbal ability. The data were analysed using SPSS and R, the Lavaan package [51].

A robust maximum likelihood estimator was used to account for the potential non-normal distribution of each variable where variables were continuous. For ordinal variables, diagonally weighted least squares estimation (WLSMV) was used. To evaluate whether the models fit the data, three recommended methods were used: the *root mean square of approximation* (RMSEA) with < 0.05 indicative of good model fit and values between 0.05 and 0.08 as adequate model fit [52]; the *comparative fit index* (CFI); and the Tucker-Lewis index (TLI), for which values above .95 are considered as a good fit [53]. Where two or more models met all three criteria, the simplest model that was most strongly supported by theory for further analyses was selected. Covariates (e.g., gender, age, education, English as native language and verbal ability) were treated as separate variables. In line with recommendations for individual differences researchers, correlations of .10 were considered as a small effect, .20 a typical (or moderate) effect and > .3 as a relatively large effect [54].

### Missing data

The percentage of missing values ranged from 0.8% (*N* = 1) for demographic variables such as education up to 7.8% for religious upbringing (*N* = 10). The only reason for missing data was participants’ nonresponse. A full information maximum likelihood (FIML) approach to missing data was adopted. This method has been shown to produce unbiased parameter estimates, standard errors and test statistics that are consistent and efficient when data is either missing at random (MAR) or missing completely at random (MCAR) [55]. To test for patterns within the missing data, Little’s Completely at Random test (MCAR) was conducted and showed that data was not systematically missing, χ^2^ (1205) = 1133.893, *p* = .928.

### Preliminary and descriptive statistics

Table A in the S1 Table shows the bivariate correlations between the item-level data of the SFT and MASC as well as the descriptive statistics for each item of the measures. For correlations between the original coding of the MASC and SFT with the new coding applied in the present study, see Table B in the S2 Table. Descriptive statistics of all summed study variables can be seen in Table 1. The SFT mental state terms data were normally distributed, with one participant using no mental state terms at all (.8%) and two participants (1.6%) using 15 mental state terms to describe the actions in the video clips. There were no ceiling or floor effects for response appropriateness data, with no participant scoring 0 and only 4 participants achieving full scores (3.1%). For the MASC mental state terms data, 3 participants made only two mental state references (2.3%), and one participant made 75 mental state references (.8%) whilst the second highest number of MST was 51 (*N* = 1, .8%). The data were normally distributed. In terms of appropriateness, there were no ceiling or floor effects, with no participant scoring 0, one participant scoring 1 (.8%), and three participants scoring 2 (2.3%). No participant achieved full scores, 3 participants scored 17, (2.3%), and a further 3 participants achieved 16 across all items of the mask. Skewness and kurtosis of all study measures, except for the social network size measure, were within the acceptable ranges of -3 and +3 for skewness and -10 to +10 for kurtosis. The social network size measure was positively skewed due to an outlier (one participant indicated 110 social contacts, whilst the second highest number was 43).

**Table 1:** Descriptive statistics for each study measure.

	M (SD)	Min	Max	Range	Skewness (SE)	Kurtosis (SE)	*N*
IDQA	68.52 (25.63)	18	154	136	.545 (.214)	.564 (.425)	128
SFT Original	8.32 (1.83)	1	12	11	-1.173 (.217)	2.47 (.430)	125
MASC Correct	7.29 (1.46)	1	10	9	-.841 (-.841)	2.67 (.425)	128
MASC Exc	1.35 (.98)	0	5	5	.699 (.214)	1.04 (.425)	128
MASC Less	.95 (.92)	0	6	6	1.56 (.214)	5.83 (.43)	128
MASC No	.414 (.682)	0	3	3	1.83 (.214)	3.51 (.43)	128
MASC APP	9.25 (3.45)	1	17	16	.162 (.217)	-.16 (.430)	125
SFT APP	5.63 (2.13)	1	10	9	-.040 (.219)	-.58 (.435)	122
MASC MST	18.54 (10.55)	2	75	73	1.94 (.217)	6.7 (.430)	125
SFT MST	7.21 (3.05)	0	15	15	.463 (.219)	-.125 (.435)	122
Loneliness	2.36 (.75)	1	4	3	-.137 (.214)	-.823 (.425)	128
Autho	2.62 (.71)	1.17	4.17	3	.178 (.215)	-.691 (.427)	127
Extraversion	3.97 (1.50)	1.50	6.50	5	-.086 (.214)	-1.111 (.425)	128
Agree	5.09 (1.03)	2.50	7	4.5	-.301 (.214)	-.612 (.425)	128
Conscien	5.39 (1.14)	2	7	5	-.889 (.214)	.437 (.425)	128
Emo Stability	3.77 (1.31)	1	7	6	.105 (.214)	-.228 (.425)	128
Openness	5.04 (1.16)	2	7	5	-.327 (.214)	-.508 (.425)	128
WASI	29.20 (2.91)	22	35	13	-.488 (.221)	-.098 (.438)	120
MRM	66.12 (7.63)	47	89	42	-.098 (.217)	.326 (.430)	125
Rel Practice	1.19 (1.28)	0	5	5	1.18 (.216)	.925 (.428)	126
Rel Belief	2.28 (1.79)	0	5	5	.370 (.216)	-1.31 (.428)	126
Rel Affiliation	.428 (.497)	0	1	1	.292 (.216)	-1.946 (.428)	126
Rel Upbringing	.423 (.496)	0	1	1	.313 (.223)	-1.935 (.442)	118
Social Net Size	18.64 (12.03)	1	110	109	3.668 (.214)	25.303 (.425)	128
Ad Net Size	8.08 (1.77)	2	15	13	-.835 (.214)	2.404 (.425)	128
Emo Ad Net Size	29.52 (7.67)	10	51	41	-.087 (.214)	-.114 (.425)	128

*Note*. M = mean, SD = Standard Deviation, Min = observed minimum value, Max = observed maximum value, SE = Standard Error, N = number, IDQA = Anthropomorphism; IDQA-NA = No Anthropomorphism Scale, SFT = Silent Film Task, MASC = Movie for the Assessment of Social Cognition, Exc = Excessive Mindreading (MCQ), Less = Less Mindreading (MCQ), No = No Mindreading (MCQ), APP = Appropriateness, MST = Mental State Terms, Autho = Authoritarianism; Agree = Agreeableness; Conscien = Conscientiousness; Emo Stability = Emotional Stability; Openness = Openness to Experience, WASI = Verbal Ability, MRM = Mindreading Motivation, Rel = Religious, Net = Network, Emo = Emotional, Ad = Advice.

### Main analysis

**Fig 1:**
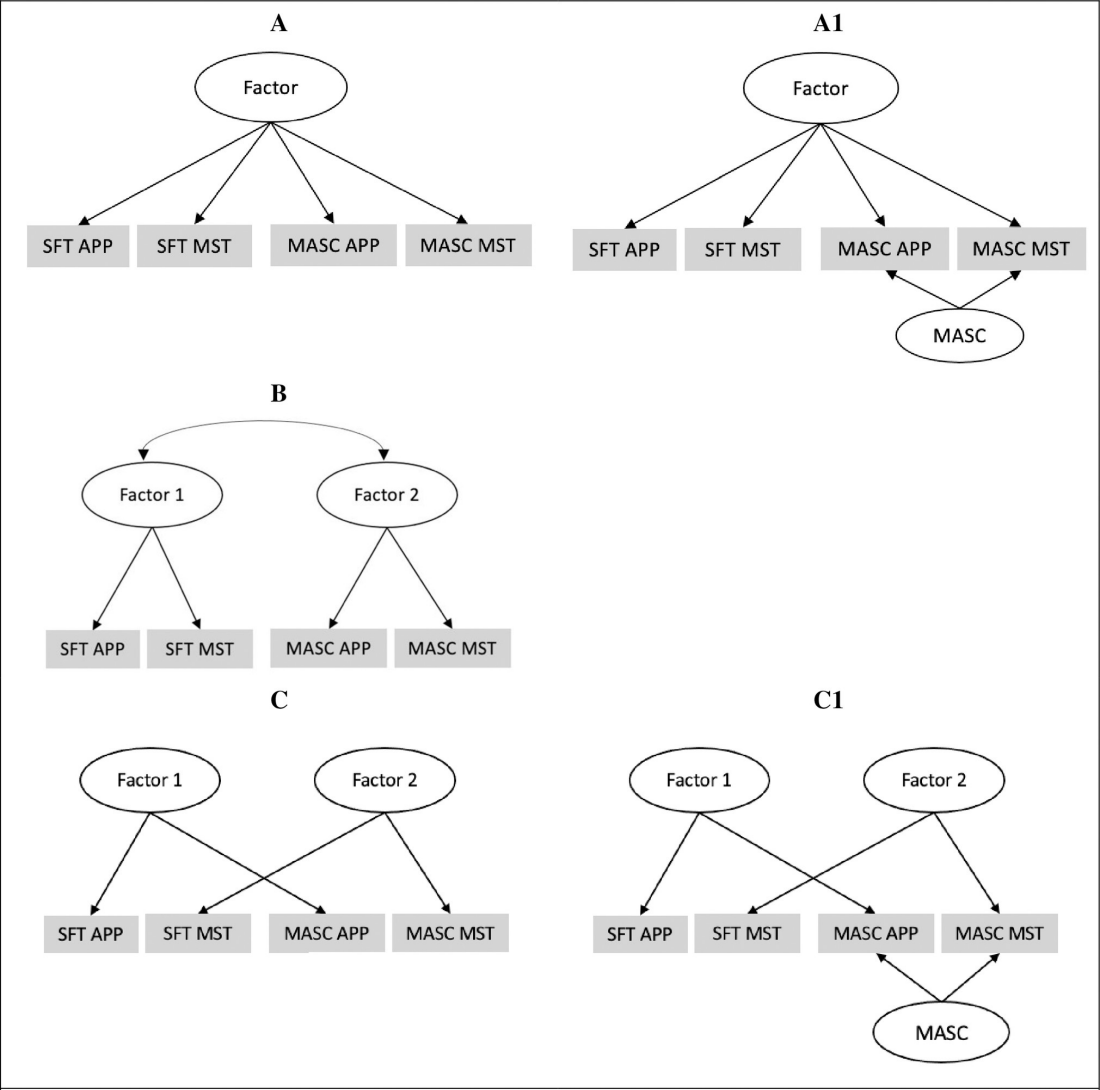
Schematic representation of the models that were tested to investigate the best fit for the item-level data of the SFT (5 items) and MASC (10 items), with each item being associated with two unique scores; a mindreading response appropriateness score and a frequencies of MST score (making 32 individual scores). A = One-Factor model where frequencies of MST and response appropriateness load onto one factor, regardless of task; 1 = Method factors due to the different tasks are accounted for (MASC); B = Two-Factor / Two-Task Model where both constructs (i.e., MST and response appropriateness) coded from the SFT load onto one factor (representing performance on the SFT) and both constructs coded from the MASC load onto a separate factor to test if shared task characteristics drove individual differences in response appropriateness and MST; C = Two-Factor model where response appropriateness and MST load onto separate factors.

The latent factor structure of mindreading (i.e., whether response appropriateness and frequencies of MST are separable or overlapping) was examined. A schematic overview of the different models that were tested is shown in Fig 1 and the summary statistics of the different models are shown in Table 1. Both a one-factor (see model A.123) and two-factor (see model C.23) model provided an acceptable fit for the data (One factor model: RMSEA = .032, CFI = .937, TLI = .927, Factor loadings = .115 - .679; Two factor model: RMSEA = .031, CFI = .943, TLI = .933, Factor loadings = .132 - .733). Additionally, the latent factors of response appropriateness and frequencies of MST were significantly positively correlated, r = .75 (see Table 2). Therefore, in terms of our first criterion for judging the value of coding MST and appropriateness separately versus in a single construct, although indices were marginally in favour of a two-factor solution, there is no clear evidence in favour of a one- or two-factor model. The second criterion was therefore considered. Specifically, the associations between the resulting factors from these models were compared with the outcome variables.

**Table 2:** Bivariate correlations between all variables of interest.

Variables	1	2	3	4	5	6	7	8	9	10	11	12	13	14	15	16	17	18	19	20
1. LFA																				
2. LF M	.745 **																			
3. LFMA	.756**	.962**																		
4. MRM	.208*	.169	.086																	
5. Lone	-.014	-.036	-.040	-.110																
6. Anthro	.178*	.147	.137	.132	.233**															
7. Authori	.068	.064	.049	-.194*	.067	.026														
8. LF Rel	-.152	.044	.028	-.091	.070	.078	.157													
9. NetS	-.039	-.043	-.039	.112	-.139	-.078	.009	-.008												
10. AdS	.053	-.026	-.066	.112	-.139	.112	.009	.064	.156											
11. AdE	.105	.059	.033	-.121	-.353**	.092	.079	.080	.178*	.722**										
12. Edu	-.070	.160	.169	.035	.017	.037	.046	-.033	-.006	-.095	-.007									
13. Gen	.104	.058	.036	.090	.042	.197*	-.055	-.011	.054	-.039	.133	.137								
14. Nat	-.030	.112	.089	-.018	.202*	.096	.072	.134	-.150	-.032	-.017	.210*	-.083							
15. Age	-.024	.181*	.206*	.037	-.078	-.056	-.074	-.079	-.080	-.041	-.090	.551**	.030	.137						
16. VA	.206*	.097	.168	.2312*	-.003	.001	-.172	.051	.188*	-.009	-.038	.058	-.009	-.093	.002					
17. Extra	.025	-.014	-.042	.147	-.294**	-.064	-.153	-.095	.231*	.004	.042	-.040	-.015	-.161	.074	-.021				
18. Agree	.194*	.112	.159	.124	-.076	.211*	-.014	.135	.113	.050	.176*	.063	.196*	-.037	-.014	.159	-.039			
19. Cons	.051	.072	.108	.131	-.081	.029	.147	.082	.098	-.038	.128	.004	.124	-.039	-.179*	-.020	-.019	.107		
20. Emo	-.090	-.049	-.020	-.251**	-.382**	-.224*	.065	.119	.024	.057	.229**	.047	-.056	.095	.110	-.021	.204*	.181*	.082	
21. Open	.037	.050	.016	.060	-.056	.027	-.155	.169	.119	-.004	-.014	.139	.181*	.129	.094	-.042	.312**	.155	-.033	.137

*Note*. **p < .01.

*p < .05. LF = Latent Factor, A = Appropriateness, M = MST, MRM = Mindreading Motivation, Lone = Loneliness, Authori = Authoritarianism, Rel = Religiosity, Up = Upbringing, A = Affiliation, P = Practice, B = Belief, Net = Social Network, S = Size Ad = Advice Network, E = Emotional Closeness, Edu = Education, Gen = Gender / Sex, Nat = English Native Language, VA = Verbal Ability, Extra = Extraversion, Agree = Agreeableness, Cons = Conscientiousness, Emo = Emotional Stability, Open = Openness to Experience.

Next, associations between the resulting factors from these models and the social outcome variables were compared. Table 2 shows the bivariate correlations between all study variables. The latent factors for response appropriateness and frequencies of MST were strongly positively correlated, indicating that there was a large amount of shared variance between both constructs. Although there was a small significant association between response appropriateness and mindreading motivation, frequencies of MST and mindreading motivation were not significantly correlated. Anthropomorphism was weakly positively associated with response appropriateness and negatively with loneliness. Authoritarianism was weakly negatively associated with mindreading motivation.

Table 3 displays the summary statistics for the structural equation models that were estimated to test how either response appropriateness and frequencies of MST, separately as part of a two-factor model, or jointly, as part of a one-factor model, related to other constructs of interest. Each outcome variable was separately regressed onto these two latent factors. To control for potential confounding variables, the outcome variables were regressed onto each latent factor and education, gender, age, whether they spoke English as their native language, verbal ability and personality traits. Covariates were permitted to correlate with each other in each model.

**Table 3:** Summary statistics of different models to assess whether response appropriateness and frequencies of MST are separable or overlapping.

		RMSEA	CFI	TLI	Factor Loadings	Scores from Items no longer sig. loading onto Latent Factor			
Good Fit Indices		< .08	>.90	>.90	(range)	SFT		MASC	
**Model**						MST	APP	MST	APP
***One-Factor***	A	.061	.814	.792	.141 - .664				
Bi-Factor	A1	.055	.861	.837	.129 - .715		Q3	-	Q2, Q24
	A12	.049	.856	.835	.136 - .693	Q1, Q2	Q3	-	Q2,
	A.132	.032	.937	.927	.115 - .679	Q1, Q2	Q1, Q3	-	Q2, Q24
***Two-Factor***									
MASC vs SFT	B	.084	.636	.608	.153 - .743	Q2	-	-	-
Bi-factor	B2	.049	.886	.866	.157 - .663	Q2	-	-	Q1, Q2
	B24	.044	.884	.866	.171 - .690	Q2	Q1	-	-
MST vs APP	C	.058	.836	.816	.189 - .705				
Bi-factor	C1	.052	.872	.849	.129 - .659	-	-	-	Q2, Q24, Q34, Q39
	C12	.047	.867	.847	.160 - .654	Q1, Q2	-	-	Q2, Q24
	C.23	.031	.943	.933	.132 - .733	Q1, Q2, Q4,	Q1	-	Q2, Q24, Q34, Q39

Note. A = One-factor Model, B = Two-factor Model (MASC vs SFT), C = Two-factor Model (MST vs App), 1 = method factor (MASC scores), 2 = controlled for demographic variables (age, gender, education, verbal ability, personality, 3 = model fit based on most significant modification indices, 4 = method factor (MST scores), RMSEA = Root Mean Square Error of Approximation; CFI = Comparative Fit Index; TLI = Tucker-Lewis Index. Model A.123 and C.23 provided the best fit to the data. Re-running model C.23 with all non-significant items removed showed similarly good model fit, CFI = .916, TLI = .903, RMSEA = .038. The original model was therefore retained to maximise available information.

**Fig 2:**
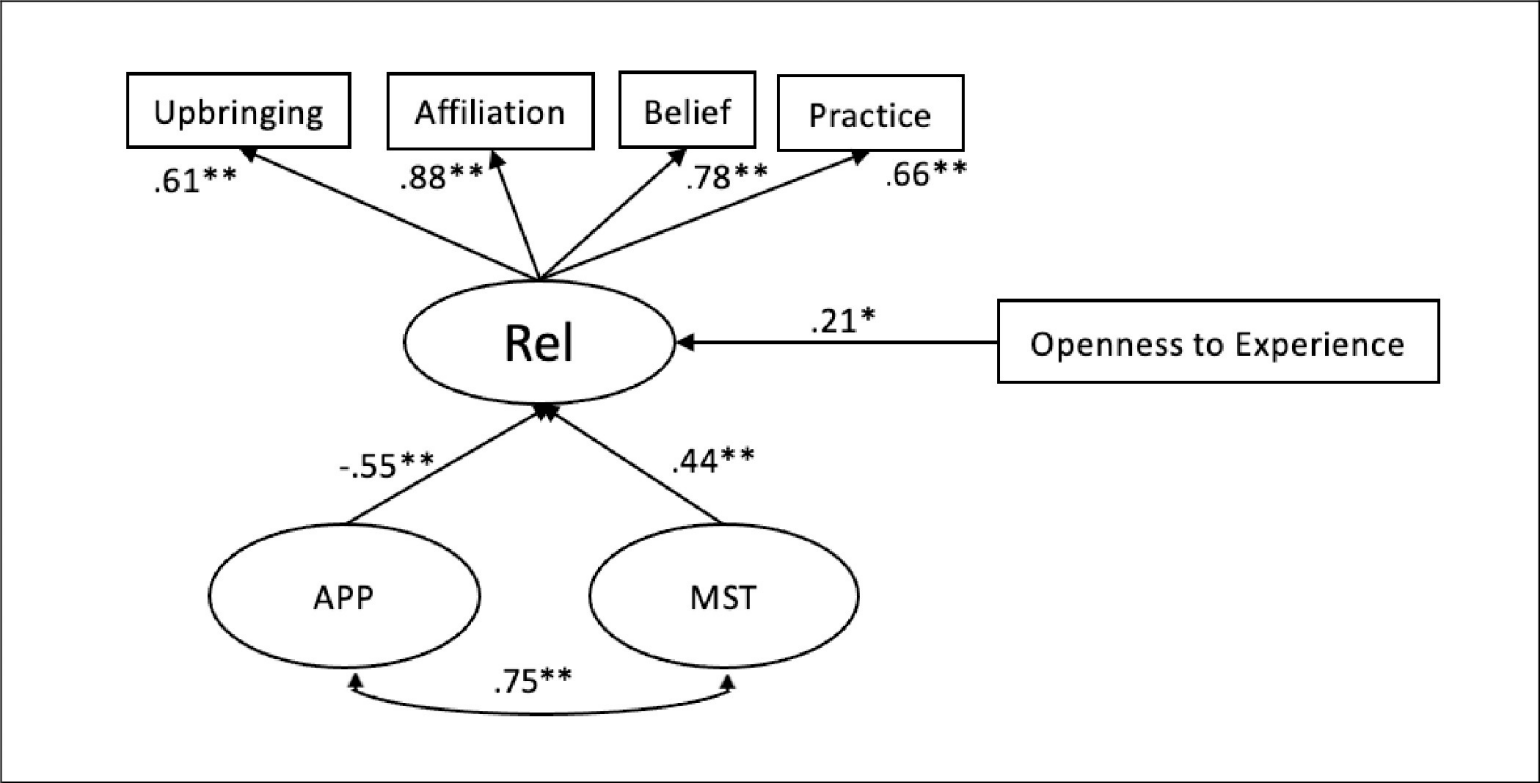
** *p* < .001, *p < .05. Simplified path diagram showing standardized estimates for statistically significant regression paths for the two-factor religiosity latent factor model (see Table 4). Covariances between predictor variables are not displayed. Non-significant variables controlled for in this model are age, gender, verbal ability, English as native language, and personality (e.g., extraversion, agreeableness, conscientiousness). Rel = Religiosity; APP = Response appropriateness; MST = Mental State Terms.

An assessment of the unique associations between mindreading motivation, frequencies of MST and response appropriateness showed that MRM was not predicted by the amount of MST people used in their responses or by the appropriateness of their responses. Likewise, a one-factor solution where frequencies of MST and response appropriateness loaded onto one factor was not a significant predictor of MRM. A one-factor solution did not predict any of the outcome variables significantly. However, for a two-factor solution there were significant, differential associations between the latent factors of frequencies of MST and response appropriateness with religiosity. MST predicted religiosity positively while greater response appropriateness was a significant negative predictor of religiosity (see Fig 2 below for a simplified path diagram of the standardized estimates for significant regression paths). This finding indicates that there was valid non-shared variance between both constructs, which might be obscured by confounding or not differentiating between the degree to which participants quantitatively engage with others’ mental states and the degree to which their responses are appropriate. In terms of our second criterion for judging the value of coding MST and appropriateness separately versus in a single construct, evidence of greater explanatory power for a two-factor solution for predicting outcomes related to mindreading was found.

**Table 4:** Regression summary statistics. Separate one- and two-factor models were run for each listed outcome variable individually.

Outcome Variable	Model		Regression Paths (standardized)	Standard Error	P—value
Mindreading Motivation	2-Factor	MST	.070	1.132	.627
		APP	.055	1.213	.705
	1-Factor	MST + APP	.088	.710	.349
Loneliness	2-Factor	MST	-.020	.103	.876
		APP	.013	.107	.919
	1-Factor	MST + APP	-.029	.066	.733
Religiosity	2-Factor	MST	.438	.133	.001*
		APP	-.532	.143	.001*
	1-Factor	MST+APP	-.049	-.046	.602
Authoritarianism	2-Factor	MST	.202	.107	.159
		APP	-.244	.114	.091
	1-Factor	MST + APP	.032	.067	.736
Social Network Size	2-Factor	MST	.039	1.827	.781
		APP	-.146	1.954	.307
	1-Factor	MST + APP	-.078	1.141	.396
Advice Network Size	2-Factor	MST	-.137	.272	.359
		APP	.124	.291	.359
	1-Factor	MST + APP	-.113	.169	.239
Emotional Closeness	2-Factor	MST	-.016	1.174	.910
		APP	.049	1.255	.689
	1-Factor	MST + APP	-.012	.730	.895
Anthropomorphism	2-Factor	MST	.040	3.820	.778
		APP	.082	4.086	.570
	1-Factor	MST + APP	.082	2.384	.375

*Note*. *p < .01. MST = Mental State Terms; APP = Response appropriateness. See S1 File for details on the formation of the religiosity latent factor.

## Discussion

In this study, we examined whether quantity and quality of mindreading were (a) separable constructs, (b) related differentially to a variety of outcome measures, and (c) accounted for by individual differences in mindreading motivation. To do this, individual differences in adults’ response “appropriateness” and their frequency of mental state terms (MST) on the Silent Film Task (SFT) and Movie for the Assessment of Social Cognition (MASC) were measured. Although response appropriateness and frequencies of MST were strongly correlated with one another, a two-factor model with frequency of MST and response appropriateness loading onto two separate but correlated factors provided greater explanatory power than a one-factor model when predicting a socially relevant outcome. Specifically, frequency of MST was positively associated with religiosity and response appropriateness was negatively associated with religiosity, whereas the one factor model did not predict any outcomes. There were no significant associations between either factor, and mindreading motivation. This provides some support for the distinctiveness of mindreading quantity (measured as frequency of MST) and mindreading quality (measured as response appropriateness) in the structure of individual differences in mindreading. These results will be discussed in more detail below.

### Evidence for a two-factor structure of mindreading

A significant current challenge for the field is to advance theoretical understanding of how mindreading abilities continue to vary in adults and to devise psychometrically robust ways to measure these individual differences [29]. Previous research has proposed that mindreading may comprise multiple component processes such as cognitive versus affective [12, 56], social-perceptual versus social-cognitive [57] or effortful versus automatic [58]. Whereas these proposals focus on the cognitive architecture of mindreading, we sought to characterise sources of variation in performance. This is relevant for any given component process, for “advanced” tasks like the MASC and SFT that combine multiple components, and for everyday use of mindreading outside of scientific settings.

Unlike many “laboratory” tasks [13, 29] the MASC and SFT are known to generate robust individual differences and to predict social outcomes, but their coding schemes combine the quality and quantity of mindreading responses into a single scale reflecting the degree to which a response is “correct”. It might be that adults’ mindreading truly varies on a single dimension. However, it is not possible to test this hypothesis when mindreading quality and quantity are baked into a unidimensional coding scheme. Based upon growing evidence that motivation contributes to individual differences in mindreading [4–6], it was hypothesised that some people might be disposed to mindread more than others, even if they were not especially successful in doing so. Therefore, mindreading quality and quantity were coded separately, and whether these factors were indeed reflected in the covariance structure of the tasks was tested.

To do so, we tested (a) whether both constructs loaded onto one or two latent factors; and (b) whether a one- or two-factor model was superior at predicting outcome variables which had previously been linked with mindreading. Evidence on the first criterion was equivocal, as both a one- and two-factor solution provided an acceptable fit for the data. Whilst response appropriateness and frequencies of MST were from the same two tasks, there was a risk that either the one-factor solution, or the large amount of shared variance between the constructs in the two-factor solution, were due to shared methods rather than similarity in the underlying constructs. However, no evidence was found for this because shared method variance was accounted for by running a two-task model, which did not provide a good fit for the data.

In relation to our second criterion, a one-factor model (representing the shared variance between MST and response appropriateness) was found to not predict any of the outcomes measured in this study. In contrast, a two-factor solution revealed significant, differential associations between the MST latent factor and response appropriateness latent factor (two-factor model) with religiosity. This finding suggests that despite substantial shared variance, response quality and quantity have explanatory power as distinct constructs. It remains important for future work to determine whether the non-shared or shared variance between MST and response appropriateness predicts other criterion variables above and beyond the ones employed here.

In summary, given the support found for both a one-factor and two-factor solution, consideration of parsimony might favour the one-factor solution. However, since the two-factor solution has superior power at predicting outcomes, we believe there are good grounds to favour the two-factor solution.

### Mindreading motivation

Given that the MASC and SFT involve mental concepts that participants should have mastered by adulthood, variation in performance could indicate differences in motivation to reason about others [5, 29, 33–36]. However, the present results indicated that self-reported motivation was not associated with either the amount of MST people used in their responses or with the appropriateness of their responses. It should be noted that minimally 13 and maximally 91 points could be achieved on the MRM scale but in the current sample, the lowest MRM score was 47, and the highest 89 (with data normally distributed between this range). This suggests that the variance of the MRM variable may have been restricted in the current study, perhaps because most of the participants were undergraduate psychology students. Likewise, a one-factor solution where frequencies of MST and response appropriateness loaded onto one factor was not associated with MRM. The present study therefore provided no evidence that frequencies of MST, and / or response appropriateness captured meaningful individual differences in self-reported mindreading motivation. However, it is important to note that the present study used a relatively brief self-report measure of mindreading motivation. It remains possible that different effects might be observed within a more diverse sample, or with more direct measures of motivation, or on tasks that assess a broader range of social motivations, including but not limited to motivation for mindreading. The origin of variation in MST remains currently unclear and future research will need to examine whether the observed lack of association between frequency of MST and mindreading motivation reflected a genuine lack of association between the two constructs.

### Social outcome variables

A one-factor mindreading factor was not uniquely associated with any of the outcome variables, but there were significant, differential associations between the latent factors of frequencies of MST and mindreading response appropriateness with religiosity. Specifically, a higher degree of MST predicted religiosity (composed of the sub-components of religious belief, affiliation, practice, and upbringing) positively, whereas greater response appropriateness was a significant negative predictor of religiosity. This means that religious participants were less likely to give appropriate (or context-sensitive) answers on two mindreading tasks and more likely to use mental state language than non-religious participants. Previous research is in line with the notion that religiosity may be negatively (or not at all) related to appropriate mentalising about other *human* minds, which differ qualitatively from supernatural agents’ minds [43, 59]. The converse positive association between frequencies of MST and religiosity is likewise in line with theoretical accounts arguing that religiosity may have emerged because of the human propensity to attribute mental states [60, 61].

The present findings highlight the potential distinctiveness of mindreading quantity (i.e., frequency of MST) and mindreading quality (i.e., response appropriateness) in the structure of individual differences in mindreading.

### Limitations and future directions

First, the measures available to investigate the constructs in the present study are limited. For instance, while a validated self-report questionnaire to measure mindreading motivation was used, participants’ scores on this measure were restricted in range. In addition, some of the covariate measures (i.e., personality, verbal ability) exhibited low internal reliability, which may have prevented the detection of relationships. Researchers often distinguish between religiosity and spirituality [62], and this distinction might be relevant to the issue of mindreading. Religion is commonly perceived as more institutionalised and dogmatic than spirituality [63, 64]. Therefore, if spirituality is less tied to religious concepts (e.g., a personal God who is concerned with each individual’s life), it may not share with religiosity its positive relation with making frequent explicit mentalistic attributions to others. Finally, it should also be noted that a large proportion of participants in the present study were female psychology students. While caution must be used when generalising the present findings to a broader population, the possibility of reduced variance in the current relatively homogeneous sample if anything reduced the likelihood that reliable factor structure for the present measures would have been found. Further research should use a non-university sample, as well as include a range of educational backgrounds and ages to be more inclusive and generalisable to a larger number of individuals.

## Conclusion

In summary, our findings are consistent with the idea that response quality and quantity may be differentiable dimensions of open-ended response mindreading tasks in adults. They demonstrate valid non-shared variance between mindreading quality and quantity, which has previously been obscured by not differentiating between the degree to which participants quantitatively engage with others’ mental states and the degree to which their responses are appropriate. Coding mindreading appropriateness and frequency of MST separately enabled us to detect a differential relationship between mindreading appropriateness and frequency of MST with religiosity, that would have been obscured with a one-factor solution. However, more research is needed to elucidate how exactly motivation relates to these components of mindreading. The present approach helps our understanding of why mindreading varies in adults who already understand basic mental state concepts of their own and others’ thoughts, beliefs and desires and may ultimately help us to understand how an adult’s propensity to effortfully engage with others’ mental states (e.g., their motivation to mindread) may interact with their ability to do so appropriately.

## Supporting information

S1 Table Table A. Bivariate correlations and descriptive statistics for the item-level data of the SFT and MASC.
(DOCX)

S2 Table Table B. Comparison original and new coding MASC and SFT.
(DOCX)

S1 File Religiosity latent factor information. (DOCX)

S2 File Coding scheme. (DOCX)

## References

[1] Richardson D. R., Green L. R., & Lago T. (1998). The relationship between perspective‐taking and nonaggressive responding in the face of an attack. *Journal of Personality*, 66, 235–256. 10.1111/1467-6494.00011

[2] Mulvey K. L., Rizzo M. T., & Killen M. (2016). Challenging gender stereotypes: Theory of mind and peer group dynamics. *Developmental Science*, 19, 999–1010. doi: 10.1111/desc.12345 26395753 PMC4808471

[3] Apperly I. A. (2012). What is “theory of mind”? Concepts, cognitive processes and individual differences. *Quarterly Journal of Experimental Psychology*, 65, 825–839. 10.1080/17470218.2012.676055 22533318

[4] Carpenter J. M., Green M. C., & Vacharkulksemsuk T. (2016). Beyond perspective-taking: Mind-reading motivation. *Motivation and Emotion*, 40, 358–374. 10.1007/s11031-016-9544-z

[5] Devine R. T., & Apperly I. A. (2022). Willing and able? Theory of mind, social motivation, and social competence in middle childhood and early adolescence. *Developmental Science*, 25, e13137. doi: 10.1111/desc.13137 34235829

[6] Contreras-Huerta L. S., Pisauro M. A., & Apps M. A. (2020). Effort shapes social cognition and behaviour: A neuro-cognitive framework. *Neuroscience & Biobehavioral Reviews*, 118, 426–439. doi: 10.1016/j.neubiorev.2020.08.003 32818580

[7] Malcolm C., Saxton T. K., McCarty K., Roberts S. G., & Pollet T. V. (2021). Extraversion is associated with advice network size, but not network density or emotional closeness to network members. *Personality and Individual Differences*, 168, 110311. 10.1016/j.paid.2020.110311

[8] Waytz A., Cacioppo J., & Epley N. (2010). Who sees human? The stability and importance of individual differences in anthropomorphism. *Perspectives on Psychological Science*, 5, 219–232. doi: 10.1177/1745691610369336 24839457 PMC4021380

[9] Wongpakaran N., Wongpakaran T., Pinyopornpanish M., Simcharoen S., Suradom C., Varnado P., et al. (2020). Development and validation of a 6‐item Revised UCLA Loneliness Scale (RULS‐6) using Rasch analysis. *British Journal of Health Psychology*, 25, 233–256. doi: 10.1111/bjhp.12404 31999891

[10] Devine R. T., & Hughes C. (2013). Silent films and strange stories: Theory of mind, gender, and social experiences in middle childhood. *Child Development*, 84, 989–1003. doi: 10.1111/cdev.12017 23199139

[11] Castelli F., Frith U., Happe F., & Frith C. D. (2001). Autism and the perception of intentionality in moving geometrical shapes. *Neuroimage*, 6, 1035. doi: 10.1016/S1053-8119(01)90009-6

[12] Schaafsma S. M., Pfaff D. W., Spunt R. P., & Adolphs R. (2015). Deconstructing and reconstructing theory of mind. *Trends in Cognitive Sciences*, 19, 65–72. doi: 10.1016/j.tics.2014.11.007 25496670 PMC4314437

[13] Warnell K. R., & Redcay E. (2019). Minimal coherence among varied theory of mind measures in childhood and adulthood. *Cognition*, 191, 103997. doi: 10.1016/j.cognition.2019.06.009 31229848

[14] Declerck C. H., & Bogaert S. (2008). Social value orientation: Related to empathy and the ability to read the mind in the eyes. *The Journal of Social Psychology*, 148, 711–726. doi: 10.3200/SOCP.148.6.711-726 19058659

[15] Oakley B. F., Brewer R., Bird G., & Catmur C. (2016). Theory of mind is not theory of emotion: A cautionary note on the Reading the Mind in the Eyes Test. *Journal of Abnormal Psychology*, 125, 818. doi: 10.1037/abn0000182 27505409 PMC4976760

[16] Abell F., Happe F., & Frith U. (2000). Do triangles play tricks? Attribution of mental states to animated shapes in normal and abnormal development. *Cognitive Development*, 15, 1–16. 10.1016/S0885-2014(00)00014-9

[17] Epley N., Waytz A., & Cacioppo J. T. (2007). On seeing human: a three-factor theory of anthropomorphism. *Psychological Review*, 114, 864. doi: 10.1037/0033-295X.114.4.864 17907867

[18] Tahiroglu D., & Taylor M. (2019). Anthropomorphism, social understanding, and imaginary companions. *British Journal of Developmental Psychology*, 37, 284–299. doi: 10.1111/bjdp.12272 30460701

[19] Baron-Cohen S., Wheelwright S., Hill J., Raste Y., & Plumb I. (2001). The “Reading the Mind in the Eyes” Test revised version: a study with normal adults, and adults with Asperger syndrome or high-functioning autism. *The Journal of Child Psychology and Psychiatry and Allied Disciplines*, 42, 241–251. 10.1017/S0021963001006643 11280420

[20] Quesque F., & Rossetti Y. (2020). What do theory-of-mind tasks actually measure? Theory and practice. *Perspectives on Psychological Science*, 15, 384–396. doi: 10.1177/1745691619896607 32069168

[21] Yeung E. K. L., Apperly I. A., & Devine R. T. (2023). Measures of individual differences in adult theory of mind: A systematic review. Neuroscience & Biobehavioral Reviews, 105481. doi: 10.1016/j.neubiorev.2023.105481 38036161

[22] Bradford E. E., Hukker V., Smith L., & Ferguson H. J. (2018). Assessing belief-attribution in adults with and without autism spectrum disorders using a computerised false-belief task. 10.1002/aur.2032 30345695

[23] Preißler S., Dziobek I., Ritter K., Heekeren H. R., & Roepke S. (2010). Social cognition in borderline personality disorder: evidence for disturbed recognition of the emotions, thoughts, and intentions of others. *Frontiers in Behavioral Neuroscience*, 4, 182. doi: 10.3389/fnbeh.2010.00182 21151817 PMC2999836

[24] Devine R. T., & Hughes C. (2019). Let’s talk: Parents’ mental talk (not mind‐mindedness or mindreading capacity) predicts children’s false belief understanding. *Child Development*, 90, 1236–1253. doi: 10.1111/cdev.12990 29115674

[25] Slaughter V., & Repacholi B. (2004). Introduction individual differences in theory of mind: What are we investigating?. In *Individual Differences in Theory of Mind* (pp. 11–23). Psychology Press. 10.4324/9780203488508

[26] Dziobek I., Fleck S., Kalbe E., Rogers K., Hassenstab J., Brand M., et al. (2006). Introducing MASC: a movie for the assessment of social cognition. *Journal of Autism and Developmental Disorders*, 36, 623–636. doi: 10.1007/s10803-006-0107-0 16755332

[27] Devine R. T., Kovatchev V., Grumley Traynor I., Smith P., & Lee M. (2023). Machine learning and deep learning systems for automated measurement of “advanced” theory of mind: Reliability and validity in children and adolescents. *Psychological Assessment*, 35 *(*2*)*, 165. doi: 10.1037/pas0001186 36689387

[28] Devine R. T., & Hughes C. (2016). Measuring theory of mind across middle childhood: Reliability and validity of the silent films and strange stories tasks. *Journal of Experimental Child Psychology*, 149, 23–40. doi: 10.1016/j.jecp.2015.07.011 26255713

[29] Qureshi A. W., Monk R. L., Samson D., & Apperly I. A. (2020). Does interference between self and other perspectives in theory of mind tasks reflect a common underlying process? Evidence from individual differences in theory of mind and inhibitory control. *Psychonomic Bulletin & Review*, 27, 178–190. doi: 10.3758/s13423-019-01656-z 31429057 PMC7000534

[30] Meins E., Fernyhough C., Johnson F., & Lidstone J. (2006). Mind‐mindedness in children: Individual differences in internal‐state talk in middle childhood. *British Journal of Developmental Psychology*, 24, 181–196. 10.1348/026151005X80174

[31] Lecce S., Ceccato I., & Cavallini E. (2019). Theory of mind, mental state talk and social relationships in aging: The case of friendship. *Aging & Mental Health*, 23, 1105–1112. doi: 10.1080/13607863.2018.1479832 30482047

[32] Carstensen L. L., Isaacowitz D. M., & Charles S. T. (1999). Taking time seriously: A theory of socioemotional selectivity. *American Psychologist*, 54(3), 165. 10.1037/0003-066X.54.3.165 10199217

[33] Apperly I. A., Riggs K. J., Simpson A., Chiavarino C., & Samson D. (2006). Is belief reasoning automatic?. *Psychological Science*, 17, 841–844. doi: 10.1111/j.1467-9280.2006.01791.x 17100782

[34] Ferguson H. J., Apperly I., & Cane J. E. (2017). Eye tracking reveals the cost of switching between self and other perspectives in a visual perspective-taking task. *The Quarterly Journal of Experimental Psychology*, 70, 1646–1660. doi: 10.1080/17470218.2016.1199716 27364567

[35] Lin S., Keysar B., & Epley N. (2010). Reflexively mind blind: Using theory of mind to interpret behaviour requires effortful attention. *Journal of Experimental Social Psychology*, 46, 551–556. 10.1016/j.jesp.2009.12.019

[36] Kouklari E. C., Tsermentseli S., & Auyeung B. (2018). Executive function predicts theory of mind but not social verbal communication in school-aged children with autism spectrum disorder. *Research in Developmental Disabilities*, 76, 12–24. doi: 10.1016/j.ridd.2018.02.015 29547763

[37] Burnside K., Ruel A., Azar N., & Poulin-Dubois D. (2018). Implicit false belief across the lifespan: Non-replication of an anticipatory looking task. *Cognitive Development*, 46, 4–11. 10.1016/j.cogdev.2017.08.006

[38] Chevallier C., Kohls G., Troiani V., Brodkin E. S., & Schultz R. T. (2012). The social motivation theory of autism. *Trends in Cognitive Sciences*, 16, 231–239. doi: 10.1016/j.tics.2012.02.007 22425667 PMC3329932

[39] Livingston L. A., Carr B., & Shah P. (2019). Recent advances and new directions in measuring theory of mind in autistic adults. *Journal of Autism and Developmental Disorders*, 49, 1738–1744. doi: 10.1007/s10803-018-3823-3 30515619 PMC6450842

[40] Bosacki S., Moreira F. P., Sitnik V., Andrews K., & Talwar V. (2020). Theory of mind, self-knowledge, and perceptions of loneliness in emerging adolescents. *The Journal of Genetic Psychology*, 181, 14–31. doi: 10.1080/00221325.2019.1687418 31701813

[41] Atran S., & Norenzayan A. (2004). Why minds create gods: Devotion, deception, death, and arational decision making. *Behavioral and Brain Sciences*, 27, 754–770. 10.1017/S0140525X04470174

[42] Boyer P. (2008). Religion: Bound to believe?. *Nature*, 455, 1038–1039. 10.1038/4551038a 18948934

[43] Vonk J., & Pitzen J. (2017). Believing in other minds: Accurate mentalizing does not predict religiosity. *Personality and Individual Differences*, 115, 70–76. 10.1016/j.paid.2016.06.008

[44] O’Reilly J., & Peterson C. C. (2014). Theory of mind at home: Linking authoritative and authoritarian parenting styles to children’s social understanding. *Early Child Development and Care*, 184, 1934–1947. 10.1080/03004430.2014.894034

[45] Urquiza-Haas E. G., & Kotrschal K. (2015). The mind behind anthropomorphic thinking: attribution of mental states to other species. *Animal Behaviour*, 109, 167–176. 10.1016/j.anbehav.2015.08.011

[46] Soper D.S. (2024). A-priori Sample Size Calculator for Structural Equation Models [Software]. Available from https://www.danielsoper.com/statcalc

[47] Goodhew S. C., & Edwards M. (2019). Translating experimental paradigms into individual-differences research: Contributions, challenges, and practical recommendations. *Consciousness and Cognition*, 69, 14–25. doi: 10.1016/j.concog.2019.01.008 30685513

[48] Taumoepeau M., Sadeghi S., & Nobilo A. (2019). Cross-cultural differences in children’s theory of mind in Iran and New Zealand: The role of caregiver mental state talk. *Cognitive Development*, 51, 32–45. 10.1016/j.cogdev.2019.05.004

[49] Gosling S. D., Rentfrow P. J., & Swann W. B. Jr (2003). A very brief measure of the Big-Five personality domains. *Journal of Research in Personality*, 37, 504–528. 10.1016/S0092-6566(03)00046-1

[50] Wechsler D. (2001). *Wechsler Test of Adult Reading*: *WTAR*. Psychological Corporation.

[51] Rosseel Y. (2012). Lavaan: An R package for structural equation modelling and more. Version 0.5–12 (BETA). *Journal of Statistical Software*, 48, 1–36. doi: 10.18637/jss.v048.i02

[52] Brown T. A. (2015). *Confirmatory Factor Analysis for Applied Research* *(*2^nd^ ed.*)*. London: The Guilford Press.

[53] Kline R. B. (2011). Convergence of structural equation modelling and multilevel modelling. doi: 10.4135/9781446268261.n31

[54] Gignac G. E., & Szodorai E. T. (2016). Effect size guidelines for individual differences researchers. *Personality and Individual Differences*, 102, 74–78. 10.1016/j.paid.2016.06.069

[55] Enders C. K., & Bandalos D. L. (2001). The relative performance of full information maximum likelihood estimation for missing data in structural equation models. Structural Equation Modelling, 8, 430–457. doi: 10.1207/S15328007SEM0803_

[56] Rossetto F., Castelli I., Baglio F., Massaro D., Alberoni M., Nemni R., et al. (2018). Cognitive and affective theory of mind in mild cognitive impairment and Parkinson’s disease: preliminary evidence from the Italian version of the yoni task. *Developmental Neuropsychology*, 43, 764–780. doi: 10.1080/87565641.2018.1529175 30299987

[57] Tager-Flusberg H., & Sullivan K. (2000). A componential view of theory of mind: evidence from Williams syndrome. *Cognition*, 76(1), 59–90. doi: 10.1016/s0010-0277(00)00069-x 10822043

[58] Apperly I. A., & Butterfill S. A. (2009). Do humans have two systems to track beliefs and belief-like states?. *Psychological Review*, 116, 953. doi: 10.1037/a0016923 19839692

[59] Zmigrod L., Rentfrow P. J., Zmigrod S., & Robbins T. W. (2019). Cognitive flexibility and religious disbelief. *Psychological Research*, 83, 1749–1759. doi: 10.1007/s00426-018-1034-3 29948184 PMC6794241

[60] Bering J. M., & Shackelford T. K. (2004). Supernatural agents may have provided adaptive social information. *Behavioral and Brain Sciences*, 27, 732–733. 10.1017/S0140525X04240171

[61] Norenzayan A., Gervais W. M., & Trzesniewski K. H. (2012). Mentalizing deficits constrain belief in a personal God. *PloS One*, 7(5), e36880. doi: 10.1371/journal.pone.0036880 22666332 PMC3364254

[62] McPhetres J., & Zuckerman M. (2018). Religiosity predicts negative attitudes towards science and lower levels of science literacy. *PloS One*, 13, e0207125. doi: 10.1371/journal.pone.0207125 30481175 PMC6258506

[63] Popp-Baier U. (2010). From religion to spirituality—Megatrend in contemporary society or methodological artefact? A contribution to the secularization debate from psychology of religion. *Journal of Religion in Europe*, 3, 34–67. 10.1163/187489209X478337

[64] Guthrie S. E. (1996). Religion: What is it?. *Journal for the Scientific Study of Religion*, 412–419. 10.2307/1386417

