# Supplementary material for: Mindreading quality versus quantity: A theoretically and empirically motivated two-factor structure for individual differences in adults’ mindreading
Source: PLoS One. 2024 Jun 25;19(6):e0305270. doi: 10.1371/journal.pone.0305270 (PMC11198895; doi:10.1371/journal.pone.0305270)
Supplement: S1 Table — Table A. Bivariate correlations and descriptive statistics for the item-level data of the SFT and MASC. (DOCX) [file pone.0305270.s001.docx]

**S1** Table A

| Table A. *Bivariate correlations and descriptive statistics for the item-level data of the SFT and MASC.* | | | | | | | | | | | | | | | | | | | | | | | | | | | | | | |
| --- | --- | --- | --- | --- | --- | --- | --- | --- | --- | --- | --- | --- | --- | --- | --- | --- | --- | --- | --- | --- | --- | --- | --- | --- | --- | --- | --- | --- | --- | --- |
| ***Variable*** | 1 | 2 | 3 | 4 | 5 | 6 | 7 | 8 | 9 | 10 | 11 | 12 | 13 | 14 | 15 | 16 | 17 | 18 | 19 | 20 | 21 | 22 | 23 | 24 | 25 | 26 | 27 | 28 | 29 | 30 |
| 1MASC_1_A |  |  |  |  |  |  |  |  |  |  |  |  |  |  |  |  |  |  |  |  |  |  |  |  |  |  |  |  |  |  |
| 2MASC_2_A | .071 |  |  |  |  |  |  |  |  |  |  |  |  |  |  |  |  |  |  |  |  |  |  |  |  |  |  |  |  |  |
| 3MASC_13_A | .016 | .122 |  |  |  |  |  |  |  |  |  |  |  |  |  |  |  |  |  |  |  |  |  |  |  |  |  |  |  |  |
| 4MASC_18_A | .072 | .195* | .246** |  |  |  |  |  |  |  |  |  |  |  |  |  |  |  |  |  |  |  |  |  |  |  |  |  |  |  |
| 5MASC_24_A | .078 | .216* | .258** | .254** |  |  |  |  |  |  |  |  |  |  |  |  |  |  |  |  |  |  |  |  |  |  |  |  |  |  |
| 6MASC_28_A | .224* | .119 | .186* | .365** | .197* |  |  |  |  |  |  |  |  |  |  |  |  |  |  |  |  |  |  |  |  |  |  |  |  |  |
| 7MASC_30_A | .155 | .067 | .042 | .146 | .064 | .320** |  |  |  |  |  |  |  |  |  |  |  |  |  |  |  |  |  |  |  |  |  |  |  |  |
| 8MASC_34_A | .133 | .038 | .178* | .125 | .162 | .190* | .155 |  |  |  |  |  |  |  |  |  |  |  |  |  |  |  |  |  |  |  |  |  |  |  |
| 9MASC_39_A | .103 | .172 | .226* | .314** | .317** | .132 | .091 | .293** |  |  |  |  |  |  |  |  |  |  |  |  |  |  |  |  |  |  |  |  |  |  |
| 10MASC_45_A | .076 | .150 | .122 | .331** | .220* | .207* | .262** | .117 | .306** |  |  |  |  |  |  |  |  |  |  |  |  |  |  |  |  |  |  |  |  |  |
| 11SFT_1_A | .129 | .090 | .186* | .094 | .142 | .036 | .063 | -.046 | .122 | .142 |  |  |  |  |  |  |  |  |  |  |  |  |  |  |  |  |  |  |  |  |
| 12SFT_2_A | .051 | .082 | .137 | .097 | .037 | .103 | .095 | .080 | .046 | .200* | .163 |  |  |  |  |  |  |  |  |  |  |  |  |  |  |  |  |  |  |  |
| 13SFT_3_A | .154 | .131 | .075 | .094 | .195* | .096 | .182* | .012 | .001 | .182* | .103 | .158 |  |  |  |  |  |  |  |  |  |  |  |  |  |  |  |  |  |  |
| 14SFT_4_A | .192* | .018 | .081 | .070 | -.066 | .216* | .164 | .027 | .080 | .143 | .231** | .154 | .232** |  |  |  |  |  |  |  |  |  |  |  |  |  |  |  |  |  |
| 15SFT_5_A | .182* | .152 | .308** | .102 | .150 | .234** | .080 | .203* | .225* | .096 | .058 | .225* | .217* | .383** |  |  |  |  |  |  |  |  |  |  |  |  |  |  |  |  |
| 16MASC_1_M | .334** | -.045 | .050 | .244** | -.041 | .063 | .103 | .235** | .201* | .130 | .038 | .037 | .026 | .240* | .174 |  |  |  |  |  |  |  |  |  |  |  |  |  |  |  |
| 17MASC_2_M | .036 | .287** | .255** | .188** | .134 | .212* | .237** | .047 | .181* | .135 | .105 | .221* | .145 | .246** | .276** | .375** |  |  |  |  |  |  |  |  |  |  |  |  |  |  |
| 18MASC_13_M | .074 | .217* | .243** | .149 | .065 | .196* | .304** | .098 | .246** | .152 | .257** | .189* | .118 | .251** | .309** | .313** | .467** |  |  |  |  |  |  |  |  |  |  |  |  |  |
| 19MASC_18_M | .018 | .210* | .322** | .721** | .361* | .288** | .310** | .242** | .358** | .416** | .111 | .208* | .007 | .064 | .171 | .275** | .371** | .334** |  |  |  |  |  |  |  |  |  |  |  |  |
| 20MASC_24_M | -.003 | .255** | .303** | .216* | .471** | .181* | .227* | .182* | .250** | .293** | .072 | .169 | .083 | .078 | .157 | .002 | .373** | .293** | .387** |  |  |  |  |  |  |  |  |  |  |  |
| 21MASC_28_M | .120 | .004 | .085 | .181* | .076 | .289** | .358** | .136 | .162 | .278** | .032 | .159 | -.020 | .212* | .218* | .377** | .450** | .472** | .381** | .394** |  |  |  |  |  |  |  |  |  |  |
| 22MASC_30_M | .162 | .010 | -.005 | .058 | .161 | .340** | .436** | .218* | .144 | .214* | .060 | .133 | .049 | .172 | .248** | .103 | .260** | .314** | .334** | .237** | .525** |  |  |  |  |  |  |  |  |  |
| 23MASC_34_M | .178* | .047 | .273** | .168 | .088 | .189** | .349** | .469** | .339** | .236** | .096 | .135 | -.003 | .218* | .360** | .337** | .325** | .442** | .390** | .330** | .464** | .385** |  |  |  |  |  |  |  |  |
| 24MASC_39_M | .130 | .153 | .209* | .392** | .257** | .304** | .213* | .137 | .562** | .322** | .200* | .231** | .014 | .237** | .241** | .239** | .402** | .370** | .436** | .412** | .292** | .314** | .381** |  |  |  |  |  |  |  |
| 25MASC_45_M | .079 | .053 | .128 | .205* | .131 | .360** | .280** | .231** | .185* | .535** | .164 | .083 | .145 | .228* | .155 | .165 | .216* | .358** | .291** | .325** | .429** | .392** | .398** | .333** |  |  |  |  |  |  |
| 26SFT_1_M | .125 | -.002 | .078 | .181* | -.041 | .112 | .076 | .026 | -.021 | .151 | .065 | .232** | -.159 | .161 | .190* | .031 | .043 | .136 | .118 | .083 | .190* | .010 | .084 | .150 | .047 |  |  |  |  |  |
| 27SFT_2_M | .035 | -.024 | -.025 | .187* | -.077 | .076 | -.018 | -.022 | -.034 | .060 | .045 | .229** | -.014 | .093 | .056 | .261** | .141 | .109 | .124 | .043 | .147 | .005 | .127 | .098 | .157 | .112 |  |  |  |  |
| 28SFT_3_M | .178* | .073 | .049 | .155 | .055 | .054 | .221* | .065 | .088 | .170 | .078 | .169 | .353** | .229** | .266** | .007 | .293** | .196* | .109 | .301** | .263** | .277** | .244** | .153 | .217* | .118 | .018 |  |  |  |
| 29SFT_4_M | .239** | .080 | .072 | .084 | .001 | .166 | .104 | .170 | .143 | .220* | .150 | .152 | .217* | .687** | .479** | .224* | .193* | .143 | .156 | .132 | .176 | .185* | .317** | .169 | .260** | .148 | .074 | .331** |  |  |
| 30SFT_5_M | .010 | .171 | .056 | -.021 | .088 | .070 | .081 | .129 | .197* | .016 | .050 | .157 | .068 | .052 | .431** | .045 | .108 | .295** | .054 | .207* | .196* | .225* | .247** | .158 | .261** | .053 | .234** | .190* | .140 |  |
| ***Descriptive Statistics*** |  |  |  |  |  |  |  |  |  |  |  |  |  |  |  |  |  |  |  |  |  |  |  |  |  |  |  |  |  |  |
| N | 128 | 127 | 128 | 127 | 127 | 126 | 126 | 126 | 126 | 126 | 126 | 126 | 127 | 125 | 125 | 128 | 128 | 128 | 127 | 127 | 126 | 126 | 126 | 126 | 126 | 126 | 127 | 128 | 125 | 125 |
| M (SD) | .96 (.62) | 1.10 (.80) | .76 (.66) | .95 (.66) | .79 (.65) | 1.13 (.78) | .98 (.65) | .75 (.65) | 1.18 (.61) | .63 (.70) | 1.06 (.82) | 1.42 (.64) | 1.08 (.70) | .85 (.72) | 1.18 (.69) | 1.09 (1.35) | 2.20 (1.79) | 2.27 (2.13) | 2.02 (1.47) | 2.11 (1.64) | 1.61 (1.61) | 1.55 (1.40) | 2.14 (1.92) | 2.37 (1.57) | 1.22 (1.48) | .94 (1.11) | .98 (.70) | 1.88 (1.10) | 1.40 (1.22) | 1.98 (1.22) |
| Skew | .02 | -.19 | .29 | .05 | .22 | -.243 | .023 | .299 | -.120 | .645 | -.104 | -.641 | -.108 | .235 | -.253 | 2.14 | 3.37 | 1.75 | .65 | 1.11 | 1.92 | 1.58 | 1.14 | 1.17 | 1.31 | 1.24 | .16 | .63 | .82 | 1.04 |
| Kurtosis | -.35 | -1.39 | -.73 | -.71 | -.66 | -1.32 | -.59 | -.71 | -.44 | -.75 | -1.52 | -.55 | -.92 | -1.03 | -.91 | 8.54 | 20.21 | 4.48 | .44 | 1.81 | 5.56 | 4.11 | 1.24 | 2.21 | 1.06 | .93 | -.51 | .26 | .24 | 1.80 |
| Range | 0 - 2 | 0 – 2 | 0 – 2 | 0 – 2 | 0 – 2 | 0 – 2 | 0 – 2 | 0 – 2 | 0 – 2 | 0 – 2 | 0 – 2 | 0 – 2 | 0 – 2 | 0 – 2 | 0 – 2 | 0 – 9 | 0 - 15 | 0 - 12 | 0 - 7 | 0 - 8 | 0 - 10 | 0 - 8 | 0 - 9 | 0 - 9 | 0 – 6 | 0 - 4 | 0 - 3 | 0 - 5 | 0 - 5 | 0 - 7 |
| Note. *Note.* ***p* < .01. **p* < .05. SFT = Silent Film Task, MASC = Movie for the Assesment of Social Cognition, A = Appropriateness, M = Mental State Terms, N = Number, M = Mean, SD = Standard Deviation, SE = Standard Error | | | | | | | | | | | | | | | | | | | | | | | | | | | | | | |
