# Supplementary material for: Mindreading quality versus quantity: A theoretically and empirically motivated two-factor structure for individual differences in adults’ mindreading
Source: PLoS One. 2024 Jun 25;19(6):e0305270. doi: 10.1371/journal.pone.0305270 (PMC11198895; doi:10.1371/journal.pone.0305270)
Supplement: S2 Table — Table B. Comparison original and new coding MASC and SFT. (DOCX) [file pone.0305270.s002.docx]

**S2** Table B

| Table B. Comparison original and new coding MASC and SFT. | | | | | | | | | | | | | |  |  |
| --- | --- | --- | --- | --- | --- | --- | --- | --- | --- | --- | --- | --- | --- | --- | --- |
| Variable | 1 | 2 | 3 | 4 | 5 | 6 | 7 | 8 | 9 | 10 | 11 | 12 | 13 | 14 | 15 |
| 1SFT_App_  Total_Score (summed) |  |  |  |  |  |  |  |  |  |  |  |  |  |  |  |
| 2MASC_App_  Total_Score (summed) | .361** |  |  |  |  |  |  |  |  |  |  |  |  |  |  |
| 3SFT_MST_ Total_Score (summed) | .549** | .275** |  |  |  |  |  |  |  |  |  |  |  |  |  |
| 4MASC_MST_  Total_Score (summed) | .384** | .633** | .429** |  |  |  |  |  |  |  |  |  |  |  |  |
| 5 SFT_Original_ Total_Score (summed) | .210* | .285** | .297** | .349** |  |  |  |  |  |  |  |  |  |  |  |
| 6 MASC_Original_Total_ Correct ToM | -.017 | .213* | -.077 | .077 | .223* |  |  |  |  |  |  |  |  |  |  |
| 7 MASC_Original Total_Excessive ToM | -.086 | -.144 | .123 | -.089 | -.064 | -.641** |  |  |  |  |  |  |  |  |  |
| 8 MASC_Original_Total_ Less ToM | -.012 | -.133 | -.087 | -.032 | -.275** | -.566** | -.065 |  |  |  |  |  |  |  |  |
| 9 MASC_Original_Total_ No ToM | .180* | -.061 | .111 | .012 | .007 | -.446** | .016 | -.051 |  |  |  |  |  |  |  |
| 10 MST Latent Factor (MASC & SFT combined) | .494** | .400** | .595** | .915** | .332** | -.030 | -.022 | .004 | .092 |  |  |  |  |  |  |
| 11 App_Latent Factor (MASC & SFT combined) | .784** | .574** | .591** | .656** | .324** | -.028 | -.059 | .005 | .139 | .745** |  |  |  |  |  |
| 12 One-factor LF (MASC & SFT combined) | .470** | .635** | .533** | .980** | .363** | .057 | -.085 | -.048 | .065 | .945** | .734** |  |  |  |  |
| 13 MST Latent Factor (based on SFT only) | .749** | .369** | .805** | .428** | .065 | -.070 | .044 | -.050 | .154 | .566** | .753** | .525** |  |  |  |
| 14 APP Latent Factor (based on SFT only) | .840** | .379** | .728** | .423** | .113 | -.068 | .013 | -.029 | .166 | .560** | .841** | .516** | .966** |  |  |
| 15 MST Latent Factor (based on MASC only) | .364** | .595** | .431** | .984** | .095 | .063 | -.093 | -.033 | .046 | .920** | .648** | .983** | .407** | .399** |  |
| 16 APP Latent Factor (based on MASC only) | .330** | .902** | .279** | .777** | .099 | .203* | -.188* | -.108 | -.015 | .543** | .528** | .775** | .356** | .351** | .758** |
