# Supplementary material for: Mindreading quality versus quantity: A theoretically and empirically motivated two-factor structure for individual differences in adults’ mindreading
Source: PLoS One. 2024 Jun 25;19(6):e0305270. doi: 10.1371/journal.pone.0305270 (PMC11198895; doi:10.1371/journal.pone.0305270)
Supplement: S1 File — (DOCX) [file pone.0305270.s003.docx]

**S3** Religiosity Latent Factor Information

The latent factor structure of the four religiosity indicators was investigated. There were moderate to strong positive correlations between each of the four indicators (see Table 1). The fit of a one-factor solution in which each of the four religiosity indicators was loaded onto one single latent factor was examined. This model provided excellent fit to the data, χ^2^ (11) = 8.413, *p* = .676, CFI = 1.0, TLI = 1.027, RMSEA = 0 (CI = 0 - .078). All items loaded onto the single religiosity factor with standardized loadings ranging from .65 (religious upbringing) to .87 (religious affiliation), all *ps* < .001. When age and gender, education, verbal ability and whether participants had English as their native language was controlled for, model fit was still excellent: χ^2^ (11) = 8.766, *p* = .643, CFI = 1.0, TLI = 1.021, RMSEA = .0 (CI = 0 = .077).
