# Supplementary material for: Mindreading quality versus quantity: A theoretically and empirically motivated two-factor structure for individual differences in adults’ mindreading
Source: PLoS One. 2024 Jun 25;19(6):e0305270. doi: 10.1371/journal.pone.0305270 (PMC11198895; doi:10.1371/journal.pone.0305270)
Supplement: S2 File — (DOCX) [file pone.0305270.s004.docx]

**S4. Supplementary Information Coding Scheme**

General information: Answers should be coded in terms of their quantity (i.e., word count and number of articulated mental states) and their quality (i.e., appropriateness given the task requirements. Each participant should receive two distinct scores – a quantity and a quality score per item.

**QUANTITY**

| Category | Rule |
| --- | --- |
| Total Word Count | - Record the total number of words contained within a response. |
| Total MST Words | - Record the total number of explicit mental state words (i.e., desire, cognition and emotion terms) contained within a response. Simply “trying to” does not count as a mental state term. References to participant’s own mental states (e.g., I don’t know, I think) should be recorded under “MST-own". See MST list* below for more detail. |

**Sample List of Mental-State Terms**

| **Desire** | **Emotion** | **Cognitive** | **Fixed Expressions** |
| --- | --- | --- | --- |
| Want  Like (not as a preposition)  Love  Dream  Hope  Wish  Keen on  Prefer  Care  Desire  Favourite  Need  Invite  Propose  Suggest  Offer  Rather  Fancy | Happy  Angry  Sad  Upset  Excited  Frightened  Worried  Fed Up  Pleased  Proud  Scared  Cross  Fun  Grumpy  Guilty  Irritated, Hassled,  Nervous,  Proud  (un)comfortable  Feel  Uneasy  Aversive  Insecure  Concerned  Apprehensive | Think  Believe  Know  Wonder  Expect  Pretend  Forget  Remember  Imagine  Suppose (not ‘should’)  Guess  Lie  Cheat  Bet  Assume  Idea  Secret  Clever  Mistake  Plan  Realise  Notice  Understand  Opinion  Persuade  Agree  Convince  Doubtful Indecisive | “To look for” (desire, in the context of wanting something)   “Fed up” (emotion)  “To come up with” (cognitive)  “To figure out” (cognitive)  “To act out” |

## ***Appropriateness***

**QUALITY**

General information: Decide how appropriate an answer is (regardless of the quantity of words or MST). Each answer should be scored into one of three possible ordinal categories (e.g., 0,1,2) depending on how accurately it reflects the sequence. Answers are not scored down for spelling/ grammar. If participants give several alternative responses the most appropriate alternative should be scored.

**Appropriateness Scale (for the SFT and MASC):**

**General Guidance for Silent Film Task and Triangles Task**

| Category | Score | Rule |
| --- | --- | --- |
| Uninterpretable | -99 | - An answer that cannot be classified in terms of its appropriateness as it is not understandable (i.e., “I don’t know, missing, unintelligible, containing 3 or less words) |
| Appropriate | 2 | - The answer is appropriately related to the question asked. Sufficiently detailed description that enables reconstruction of important* elements of the sequence based on the response. Includes reference to *both* interacting entities. Does not include speculation which cannot be derived from the clip. |
| Partially appropriate | 1 | - Description related to sequence, but imprecise or incomplete. Some rudimentary understanding of the reasons for characters’ actions without reaching full understanding. Might only focus on one interaction partners’ behaviour and include speculation which cannot be derived from the clip. |
| Inappropriate | 0 | - Misunderstanding of the question, nonsensical descriptions, wrong descriptions, focus on minor aspect of sequence, incoherent (reader unable to reconstruct clip). |

| Category | Score | Rule |
| --- | --- | --- |
| Uninterpretable | -99 | - An answer that cannot be classified in terms of its appropriateness as it is not understandable (i.e., “I don’t know, missing, unintelligible, containing 3 or less words) |
| Appropriate | 2 | - The answer is appropriately related to the question asked. Sufficiently detailed description that enables reconstruction of important* elements of the sequence based on the response. Includes reference to *both* interacting entities. Does not include speculation which cannot be derived from the clip. |
| Partially appropriate | 1 | - Description related to sequence, but imprecise or incomplete. Some rudimentary understanding of the reasons for characters’ actions without reaching full understanding. Might only focus on one interaction partners’ behaviour and include speculation which cannot be derived from the clip. |
| Inappropriate | 0 | - Misunderstanding of the question, nonsensical descriptions, wrong descriptions, focus on minor aspect of sequence, incoherent (reader unable to reconstruct clip). |

**MASC_Q1 (1)**

| Category | Score | Examples |
| --- | --- | --- |
| Appropriate  **- *both (a) and(b) need to be fulfilled; (c) cannot not be fulfilled** | 2 | **(a) Michael visits unexpectedly and / OR compliments Sandra**  **(b) Sandra’s reaction to compliment (i.e., feels uncomfortable, surprised). Can be indirect via awareness that the compliment appeared inappropriate in the context of the interaction (i.e., Michael was uninvited, Sandra just asked a question but was complimented, Michael is creepy)**  **(c) Does *not* mention that Sandra is pleased, thankful, or equally returns Michael’s compliment**   - Michael went to Sandra's house unannounced. He then started to compliment her even though Sandra had asked what he was doing there - The man has come to see the woman after not seeing each other for a while, and he comments on her changed appearance. Possibly uninvited and unwanted as she seemed uncomfortable. - Michael came into Sandra's house and started to complement her. Sandra look flustered and a bit perturbed but tried to be nice as Michael was a bit pushy with his talking. - Michael went to Sandra's house unannounced. He then started to compliment her even though Sandra had asked what he was doing there |
| Partially appropriate  **-Either (a) or (b) need to be fulfilled; (c) cannot be fulfilled** | 1 | **(a) Michael visits unexpectedly and / OR compliments Sandra**  **(b) Sandra’s reaction to compliment (i.e., feels uncomfortable, surprised). Can be indirect via awareness that the compliment appeared inappropriate in the context of the interaction (i.e., Michael was uninvited, Sandra just asked a question but was complimented, Michael is creepy)**  **(c) Does *not* mention that Sandra is pleased, thankful, or equally returns Michael’s compliment**   - The women unexpectedly answers the door to Michael who compliments her hair. - Michael entered the house where Sandra lives. They greet each other rather uncomfortably and exchange pleasantries. Michael asks Sandra if she got a new haircut which she confirms. - Michael is complimenting Sandra. - he arrives at her house unexpectedly and compliments her new hairstyle |
| Inappropriate  **-Neither (a) not (b) are fulfilled; (c) can (but does not have to) be fulfilled** | 0 | **(a) Michael visits unexpectedly and / OR compliments Sandra**  **(b) Sandra’s reaction to compliment (i.e., feels uncomfortable, surprised). Can be indirect via awareness that the compliment appeared inappropriate in the context of the interaction (i.e., Michael was uninvited, Sandra just asked a question but was complimented, Michael is creepy)**  **(c) Does *not* mention that Sandra is pleased, thankful, or equally returns Michael’s compliment**   - Sandras old friend has come to her house after a long time and they have reunited. - it is mike's first visit to sandra's house - Susan perhaps surprises Michael. he then notices a change in her hairstyle and compliments her for which she thanks him. - a man showed up at an old friends house - Sandra is seeing Michael after a long while at her place - The male is visiting his friend. - One of the girls opens the door and there is a man stood there. They ask each other how they are and he compliments her hair. - He comes to meet her at her house - Micheal turned up to Sandras house I'm not sure if she knew that he was coming and then they begin to complement each other as if they haven't seen each other in a while |

**MASC_Q2 (2)**

| Category | Score | Examples |
| --- | --- | --- |
| Appropriate  **- *both (a) and(b) need to be fulfilled** | 2 | - **Sandra wants to meet Michal together with other people (i.e., feels uncomfortable; unhappy about Michael cancelling group-meeting)** - **Michael has contrary wishes, he wants to see her alone (e.g., persuading her). Participant needs to be explicit that he wants to meet Sandra alone – it is not enough to say he wants to play tennis instead of having dinner.** - The man is trying to persuade Sandra to go and play tennis with him but she wants to have dinner with other people instead. - Micheal and Sandra talked about a past meeting at which Sandra met Cliff. She wanted to hang out with him again however, Micheal suggested that they should go out alone. - the woman thought they were meeting up as a group not just the two of them and she feels uncomfortable |
| Partially appropriate  **-Either (a) or (b) need to be fulfilled** | 1 | 1. **Sandra wants to meet Michal together with other people (i.e., feels uncomfortable; unhappy about Michael cancelling group-meeting)** 2. **Michael has contrary wishes, he wants to see her alone (e.g., persuading her). Participant needs to be explicit that he wants to meet Sandra alone – it is not enough to say he wants to play tennis instead of having dinner.**  - They are reminiscing about a past time and Sandra mentions she met his friend Cliff there. She suggested they were meant to have dinner together and looks a bit annoyed when he said they'll do that another time. - they are talking about a time they went out together, but she mentions his friend Chris who she may like more - Sandra and Michael talk about a trip they took together where she met his friend cliff. She proposes they all make dinner at her house but Michael wants to go for a game of tennis instead. |
| Inappropriate  **-Neither (a) not (b) are fulfilled** | 0 | 1. **Sandra wants to meet Michal together with other people (i.e., feels uncomfortable; unhappy about Michael cancelling group-meeting)** 2. **Michael has contrary wishes, he wants to see her alone (e.g., persuading her). Participant needs to be explicit that he wants to meet Sandra alone – it is not enough to say he wants to play tennis instead of having dinner.**  - Sandra tells Michael that she remembers Cliff - They continued chatting, now about Cliff. - The man and woman are remembering another night they spent together with company fondly. The woman remembers an agreement to have dinner at her house, the man inquires about a game of tennis he wants to play with her instead of dinner - she is rejecting him, because she likes cliff. - They are discussing the plans they had for that night. they are also talking about his friend Clive |

**MASC_Q13 (3)**

| Category | Score | Examples |
| --- | --- | --- |
| Appropriate  **- *both (a) and(b) need to be fulfilled** | 2 | **(a) Cliff reveals that reason for him being sad is break-up from long-term relationship and / or Cliff’s current reaction (upset, sad)**  **(b) Sandra’s intention (e.g., flirt with Cliff, fancies him) AND / OR Sandra’s reaction to Cliff’s break-up (i.e., disappointed, sad)**   - Sandra is trying to flirt with Cliff but Cliff is unaware of it as he is talking about how he is still connected to hi sex girlfriend - After cracking a joke, Sandra and Cliff seem to be more comfortable around each other. She tells him he's in a good mood unlike the time they first met at the pub. Cliff opens up and tells Sandra that he had just broken up with his girlfriend of 4 years at the time and sadly mentions that even though they were separated, he still feels so connected. |
| Partially appropriate  **-Either (a) or (b) need to be fulfilled** | 1 | **(a) Cliff reveals that reason for him being sad is break-up from long-term relationship and / or Cliff’s current reaction (upset, sad)**  **(b) Sandra’s intention (e.g., flirt with Cliff, fancies him) AND / OR Sandra’s reaction to Cliff’s break-up (i.e., disappointed, sad)**   - Sandra is trying to get to know Cliff because she fancies him. - cliff was talking to sandra about his previous relationship and how he still feels connected to his ex - sandra arranged with michael and betty that they would have dinner and invite cliff so she can flirt with him |
| Inappropriate  **-Neither (a) not (b) are fulfilled** | 0 | **(a) Cliff reveals that reason for him being sad is break-up from long-term relationship and / or Cliff’s current reaction (upset, sad)**  **(b) Sandra’s intention (e.g., flirt with Cliff, fancies him) AND / OR Sandra’s reaction to Cliff’s break-up (i.e., disappointed, sad)**   - The lady and Cliff are talking about his romantic relationships. - They talked about themselves - Michael fancies the girl but she doesn't like him back, she like Cliff. Cliff is still thinking about his ex girlfriend and the other is being dragged into this. They're all about to have dinner with each other. - Cliff tells Sandra about his break up - Sandra invites Michael, Cliff and Betty to her house for a dinner on Saturday night. At first, Cliff and Betty are not too sure whether they want to go but they are persuaded by their friends; Sandra and Michael. Cliff is then the first one to come to Sandra's house and then spends time talking with Sandra and getting to know her. - The man is talking about his ex girlfriend - Mike wanted to invite his friend cliff and Sandra Betty. Sandra doesn't like Mike, finds him overly flirty but likes Cliff so wanted to meet up with Mike so he'd invite Cliff. Mike really likes Sandra and invites cliff because she's inviting Betty, he believes it'll be a double date where Cliff can get to know Betty. |

**MASC_Q18 (4)**

| Category | Score | Examples |
| --- | --- | --- |
| Appropriate  **- *both (a) and(b) need to be fulfilled** | 2 | 1. **Michal’s intention of trying to impress Sandra with story about helping lady** 2. **Sandra’s reaction (i.e., she thinks he’s bragging, doesn’t like it)**  - Sandra seems uncomfortably by Michael's presence but Michael seems unaware. He is recalling a story from his day and seems to be bragging/trying to impress Sandra with how he was trying to be a good person and help a stranger - Sandra and Cliff were talking about his holiday and dogs and cats. They got interrupted by Michael arriving and giving her flowers, he then told a story very loudly about him helping a lady that got hit by a bike Michael is trying to flirt with Sandra. Sandra is not interested but is trying to be polite. Michael is showing off about being a hero to impress her. - Cliff is talking to Sandra about his holiday in Sweden and how his dog ran away. Sandra feels sympathetic towards him. Michael arrives and gifts flowers to a disinterested Sandra. He tells her about a story where he helped a woman in order to impress her. |
| Partially appropriate  **-Either (a) or (b) need to be fulfilled** | 1 | 1. **Michal’s intention of trying to impress Sandra with story about helping lady** 2. **Sandra’s reaction (i.e., she thinks he’s bragging, doesn’t like it)**  - he is trying to impress sandra - Michael is talking about what happened during the day to impress Sandra- he makes out as if he was a hero when the car hit the woman. - The man is bragging about helping a lady who was in an accident - Cliff and Sandra talk about her ex boyfriend, Sweden and pets. Then comes Micheal and brings Sandra flowers. He touches her and tells her a story about something that happened to him that day. However, Sandra did not seem pleased. - Michael slaps Sandra on the knee and starts talking about how he witnessed a motorcyclist hit a woman and that he helped her and yelled at the driver. Cliff looks uncomfortable and Sandra looks disinterested. |
| Inappropriate  **-Neither (a) not (b) are fulfilled** | 0 | 1. **Michal’s intention of trying to impress Sandra with story about helping lady** 2. **Sandra’s reaction (i.e., she thinks he’s bragging, doesn’t like it)**  - Michael tells everyone that he saw a cyclist hit a woman. - Mike arrives and brings Sandra flowers. He talks about helping a lady who had been knocked over. - Mike liked telling a story about an event that happened to him before he arrived at Sandra's. |

**MASC_Q24 (5)**

| Category | Score | Examples |
| --- | --- | --- |
| Appropriate  **- *both (a) and(b) need to be fulfilled** | 2 | **(a) Michal’s intention of trying to impress Sandra / show off by opening champagne bottle**  **(b) Sandra’s AND / OR Betty’s reaction to Michael’s attempt to impress them (e.g., Sandra thinks he’s showing off, Betty doesn’t like his behaviour)**   - All 4 guests sit at the dinner table, Sandra seems uncomfortable with Michael's behaviour and attitude. Michael seems very braggy and likes to be the center of attention, taking control of the situation in an attempt to impress Sandra. Betty also seems as though she does not enjoy Michael's presence or behaviour - they are all sitting at the table having the champagne and sandra is being put off by micheal being overly confident - Sandra is laughing at Michael as he's trying to impress her and Betty is like 'lol this guy'. |
| Partially appropriate  **-Either (a) or (b) need to be fulfilled** | 1 | **(a)Michal’s intention of trying to impress Sandra / show off by opening champagne bottle**  **(b)Sandra’s AND / OR Betty’s reaction to Michael’s attempt to impress them (e.g., Sandra thinks he’s showing off, Betty doesn’t like his behaviour)**   - Michael wants to impress Sarah again - Micheal is trying to impress sandra again by attempting to open the bottle of champaigne - Betty is acknowledging how annoying Michael is. - the men followed them into the kitchen, Michael tries to show off again |
| Inappropriate  **-Neither (a) not (b) are fulfilled** | 0 | **(a)Michal’s intention of trying to impress Sandra / show off by opening champagne bottle**  **(b) Sandra’s AND / OR Betty’s reaction to Michael’s attempt to impress them (e.g., Sandra thinks he’s showing off, Betty doesn’t like his behaviour)**   - They were all sitting down to drink wine, and Michael offered to open an pour it. - Michael wanted to open the champagne for them. - They all moved to the kitchen |

**MASC_Q28 (6)**

| Category | Score | Examples |
| --- | --- | --- |
| Appropriate  **- *both (a) and(b) need to be fulfilled** | 2 | 1. **Michael being sexist by suggesting cooking is a woman’s job** 2. **Bettys reaction to Michael’s remark (e.g., she gets annoyed or does not like it). This can be implicit by simply stressing that Betty suggests Michael cuts the onions, an uncomfortable task.**  - michael made a rude comment so betty made one back The women go to make the dinner and when Cliff suggests Michael should go to help, he makes a sexist remark that it's a woman's job. Betty, who took offense suggested he chops onions as an insult. - Michael makes a misogynistic comment and it annoys betty - mike makes a sexist remark about cooking being for women and so betty tells him he should cut the onions - Michael expresses his dislike for cooking. He believes it is 'ladies work'. Betty offers that he should cut onions. She is being sarcastic in her approach towards him. Michael is rude. - Michael is bitter and misogynistic. Betty is not impressed and bites back. |
| Partially appropriate  **-Either (a) or (b) need to be fulfilled** | 1 | 1. **Michael being sexist by suggesting cooking is a woman’s job** 2. **Bettys reaction to Michael’s remark (e.g., she gets annoyed or does not like it). This can be implicit by simply stressing that Betty suggests Michael cuts the onions, an uncomfortable task.**  - Sandra and Betty start making dinner, with no sardines, Micheal is misogynistic when cliff suggests he helps with the cooking - They share champagne and start making dinner. Michael makes a obviously sexist comment and says that cooking is meant for women, he is reluctant to help - They are making dinner by Michael doesn't want to help he thinks it is women's work - Michael made a sexist comment |
| Inappropriate  **-Neither (a) not (b) are fulfilled** | 0 | 1. **Michael being sexist by suggesting cooking is a woman’s job** 2. **Bettys reaction to Michael’s remark (e.g., she gets annoyed or does not like it). This can be implicit by simply stressing that Betty suggests Michael cuts the onions, an uncomfortable task.**  - Sandra has an obvious attraction for Cliff and Micheal is starting to get jealous and offended - They go back to dinner - Sandra and Betty begin the cooking and Cliff volunteers himself and Michael to help. - They start to make dinner (with no sardines - Michael tries too hard to impress Sandra but then he realises that Sandra likes Cliff. |

**MASC_Q30 (7)**

| Category | Score | Examples |
| --- | --- | --- |
| Appropriate  **- *both (a) and(b) need to be fulfilled** | 2 | **(a) Michael makes a nasty joke aimed at Betty (it is important to mention that the joke is aimed at Betty, not enough to say that Michael is generally annoying)**  **(b)Betty’s reaction (e.g., annoyed)**   - They continue to prepare dinner and Cliff is willing to help in any way. Instead of helping, Michael makes rude comments towards Betty and her eating habits. Betty is visibly offended/shocked by his statement - cliff is being helpful in the kitchen with the wine and the cutting but micheal continues to be rude and betty is getting more annoyed |
| Partially appropriate  **-Either (a) or (b) need to be fulfilled** | 1 | **(a) Michael makes a nasty joke aimed at Betty (it is important to mention that the joke is aimed at Betty, not enough to say that Michael is generally annoying)**  **(b) Betty’s reaction (e.g., annoyed)**   - cliff wants to help more with the cooking- michael asks what else goes in the sauce- when betty says 2 cups of cream he says that she would want 5 cups in it to insult her - Michael is trying to be funny by being rude about Bettys weight - Michael passive aggressively called Betty fat - Michael gets ignored and proceeds to make a nasty comment about betty |
| Inappropriate  **-Neither (a) not (b) are fulfilled** | 0 | **(a) Michael makes a nasty joke aimed at Betty (it is important to mention that the joke is aimed at Betty, not enough to say that Michael is generally annoying)**  **(b) Betty’s reaction (e.g., annoyed)**   - They finish cutting the vegetables, Cliff asks about the wine, Michael asks about the sauce - Micheal made a comment about hoe betty would use more cream than needed whilst cliff opens a bottle of wine - The girls continue to make food and Micheal continues to be annoying - Micheal is starting to be rude |

**MASC_Q35 (8)**

| Category | Score | Examples |
| --- | --- | --- |
| Appropriate  **- *both (a) and(b) need to be fulfilled** | 2 | 1. **Michael justifying his nasty jokes / comments (e.g., jokes about it)** 2. **Betty’s reaction (either that she’s questioning, not happy or starting to like him – all are logical possibilities)**  - Sandra and Cliff are getting along and having pleasant conversation. As a good friend, Betty doesn't want to interrupt the pair and so makes polite conversation with Michael although she doesn't appear to be a big fan. Michael in response jokes about his rude nature, but Betty doesn't seem amused - Michael is picking up on Betty's dislike of him and he is testing her to see what she actually thinks of him and he justifies and qualifies his behaviour in a moral self-righteous way by claiming to be honest, which he actually isn't - micheal starts to feel left out from the conversation. betty notices this and tries to make a conversation about his and cliff's friendship. wherein micheal justifies his impolite behaviour at which she giggles |
| Partially appropriate  **-Either (a) or (b) need to be fulfilled** | 1 | 1. **Michael justifying his nasty jokes / comments (e.g., jokes about it)** 2. **Betty’s reaction (either that she’s questioning, not happy or starting to like him – all are logical possibilities)**  - Michael explains why he acts the way he does. - Betty and Michael are actually getting along. - Betty has decided to be more open to Michael and give him a chance |
| Inappropriate  **-Neither (a) not (b) are fulfilled** | 0 | 1. **Michael justifying his nasty jokes / comments (e.g., jokes about it)** 2. **Betty’s reaction (either that she’s questioning, not happy or starting to like him – all are logical possibilities)**  - Betty asks Michael how long he's know Cliff - Betty and Micheal are chatting - Betty starts a conversation with Michael whilst Sandra talks to Cliff - betty and billy are talking and she is trying to get to know more about him. - Cliff and Sandra are talking. Betty asks Micheal about his friendship with cliff. - sandra and cliff are involved in conversation so betty tries to start a conversation with Michael - They are now having dinner and talking |

**MASC_Q39 (9)**

| Category | Score | Examples |
| --- | --- | --- |
| Appropriate  **- *both (a), (b) and (c) need to be fulfilled** | 2 | 1. **Sandra annoyed at Betty** 2. **Michael is bragging** 3. **Sandra is annoyed by Michael**  - sandra is annoyed at betty because she keeps forgetting the rules and she is annoyed at Michael for trying to show off - Sandra is unimpressed that Betty asked what colour she was again. They both took very average shots. Michael says its time for some action and Sandra rolls her eyes and makes a sarcastic comment - Sandra looks annoyed when Betty asks which colour she is. Betty then hits the chip backwards. Sandra then takes a turn. Its then Michael turn, where he says about how professional he is at the game. Sandra looks embarrassed and annoyed- saying that she couldn't wait to see him shoot the shot sarcastically. - Michael is showing off and Sandra is tired of Betty asking the colour and Michael's behaviour |
| Partially appropriate  **- (a), or (b) or (c) need to be fulfilled** | 1 | 1. **Sandra annoyed at Betty** 2. **Michael is bragging** 3. **Sandra is annoyed by Michael**  - They were all playing a board game and Sandra got irritated at Betty's confusion and Michael not playing properly. - Sandra is annoyed that Betty is being stupid and also hates Michael. - sandra is starting to get irritated by betty - they are playing a game. sandra seems annoyed by michaels arrogance - Cliff is trying to boast saying he is the best at the game and Sandra isn't to impressed. |
| Inappropriate  **-Neither (a), (b) or (c) can be fulfilled** | 0 | 1. **Sandra annoyed at Betty** 2. **Michael is bragging** 3. **Sandra is annoyed by Michael**  - They are playing the board game - They have started playing the game. Both Betty and Sandra have their go but they do not have the best shot. Michael then says how he is going to show them how a professional is going to play the game - They finish eating and Sandra suggests a board game to play |

**MASC_Q46 (10)**

| Category | Score | Examples |
| --- | --- | --- |
| Appropriate  **- *both (a) and(b) need to be fulfilled** | 2 | 1. **Awareness of EITHER Betty’s intention to give Sandra time alone with Cliff AND / OR Sandra’s desire to spend time alone with Cliff (can be indirect by saying that Sandra “gets” to spend time with Cliff)** 2. **Mentioning of Michael’s feelings such as Michael being disappointed /reluctant to leave / accepting of the fact that it didn’t work out with Sandra (can be indirect - e.g., the goodbye was akward, Sandra prefers him to Michael)**  - Michael is reluctant to leave as he is interested in Sandra. Sandra is relieved that Michael is leaving and that she gets to spend time with Cliff - Betty wanted to be alone with Michael. It was an awkward goodbye between Michael and Sandra. - Betty makes plans to leave with Michael to leave Sandra alone with Cliff since she knows that Sandra wants to get to know Cliff better and prefers him to Michael - Betty suggests her and Michael leave so that Sandra and Cliff can be alone together. Michael says affectionately goodbye to Sandra but she does not like him and is happy for him to leave. - Sandra was happy that Michael was leaving and she is thinking that hopefully she won't have to see him again, she is happy that she's staying alone with cliff because she likes him |
| Partially appropriate  **-Either (a) or (b) need to be fulfilled** | 1 | 1. **Awareness of EITHER Betty’s intention to give Sandra time alone with Cliff AND / OR Sandra’s desire to spend time alone with Cliff (can be indirect by saying that Sandra “gets” to spend time with Cliff)** 2. **Mentioning of Michael’s feelings such as Michael being disappointed /reluctant to leave / accepting of the fact that it didn’t work out with Sandra (can be indirect - e.g., the goodbye was akward, Sandra prefers him to Michael)**  - Michael has accepted that him and sandra will probably not date - Betty and Michael are leaving Sandra and Cliff, Michael seems slightly disappointed but accepting. - micheal is going off with betty and sandra is glad he is leaving - Betty is leaving with Micheal to give Sandra alone time with Cliff - Sandra is pleased Betty and Michael are leaving so she can be alone with Cliff - betty wants to take michael out so sandra is left alone with sliff - everyone is leaving the house but cliff is staying. betty did this so cliff and sandra have more time to spend with one another - michael realised sandra doesnt like him - Betty invited Michael out so that Sandra and Cliffe could spend time together. Michael and Betty then leave |
| Inappropriate  **-Neither (a) not (b) are fulfilled** | 0 | 1. **Awareness of EITHER Betty’s intention to give Sandra time alone with Cliff AND / OR Sandra’s desire to spend time alone with Cliff (can be indirect by saying that Sandra “gets” to spend time with Cliff)** 2. **Mentioning of Michael’s feelings such as Michael being disappointed /reluctant to leave / accepting of the fact that it didn’t work out with Sandra (can be indirect - e.g., the goodbye was akward, Sandra prefers him to Michael)**  - Betty finished the game and invited Michael for a night cap - saying goodbye after an evening of catching up - betty and michael are leaving - they are leaving - Betty is off for another drink with Micheal. Micheal likes Betty instead of Sandra now.. - Michael and Betty are going out to get a drink and Cliff is staying with Sandra. The pairs say goodnight to each other and Michael and Betty leave. - Sandra says goodbye to Betty and Michael, who are leaving together. - Betty finished the game and invited Michael for a night cap - Micheal and Betty are leaving together. - Betty and mICHALE left |

**S3**

## ***Appropriateness***

**QUALITY**

General information: Decide how appropriate an answer is (regardless of the quantity of words or MST). Each answer should be scored into one of three possible ordinal categories (e.g., 0,1,2) depending on how accurately it reflects the sequence. Answers are not scored down for spelling/ grammar. If participants give several alternative responses the most appropriate alternative should be scored.

**Appropriateness Scale (for the SFT and MASC):**

**General Guidance for Silent Film Task and Triangles Task**

| Category | Score | Rule |
| --- | --- | --- |
| Uninterpretable | -99 | - An answer that cannot be classified in terms of its appropriateness as it is not understandable (i.e., “I don’t know, missing, unintelligible, containing 3 or less words) |
| Appropriate | 2 | - The answer is appropriately related to the question asked. Sufficiently detailed description that enables reconstruction of important* elements of the sequence based on the response. Includes reference to *both* interacting entities. Does not include speculation which cannot be derived from the clip. |
| Partially appropriate | 1 | - Description related to sequence, but imprecise or incomplete. Some rudimentary understanding of the reasons for characters’ actions without reaching full understanding. Might only focus on one interaction partners’ behaviour and include speculation which cannot be derived from the clip. |
| Inappropriate | 0 | - Misunderstanding of the question, nonsensical descriptions, wrong descriptions, focus on minor aspect of sequence, incoherent (reader unable to reconstruct clip). |

Clips 1 – 5: *“What do you think happened during this clip?”*

**CLIP 1: Men hiding**

| Category | Score | Examples |
| --- | --- | --- |
| Appropriate  **- *both (a) and(b) need to be fulfilled** | 2 | **(a) Recognition that the men try to avoid the woman in *relation* to the notice they have received  (b) Understanding of the mens’ intention behind hiding (e.g., they don’t want to be found, they don’t have to face her etc. so she can’t ask for the rent.). This can be indirect.**   - so I think they looked at the notice from the landlady saying the rent for two weeks was due or it was a final notice and they heard her coming and they hid under the coats so she didn’t find them so they didn’t have to pay the rent |
| Partially appropriate  **-Either (a) or (b) need to be fulfilled** | 1 | **(a) Recognition that the men try to avoid the woman in *relation* to the notice they have received  (b) Insight into the men’s intention behind hiding (e.g., they don’t want to be found, they don’t have to face her etc. so she can’t ask for the rent.)**   - Two men were somewhere where they shouldn't be or didn't want to be seen, so hid from a maid when she came to find them. |
| Inappropriate  **-Neither (a) not (b) are fulfilled** | 0 | **(a) Recognition that the men try to avoid the woman in *relation* to the notice they have received  (b) Insight into the men’s intention behind hiding (e.g., they don’t want to be found, they don’t have to face her etc. so she can’t ask for the rent.)**   - they have somebody caught and that was on the list was rent so it seems he wanted to borrow it or yeah …. I don’t have any idea why the lady she hears some noise and she knocked on the door and they were hiding. No idea why they are hiding when the lady comes in. They expect that she comes in, of course, because they are hiding but I have no idea why or what happened there |

**CLIP 2: Harold Van**

| Category | Score | Example |
| --- | --- | --- |
| Appropriate  **- Both (a) and(b) need to be fulfilled** | 2 | **(a) Description of both Harold’s (sits in the van) and the driver’s (drives away) behaviour.   (b) Recognition that the driver did not know (e.g., pay attention, realise) that Harold was in the van. This can be implicit via stressing that the driver was deaf in relation to him driving away.**   - The driver of the clean towels van was hard of hearing, and hence didn't realise or hear that someone had perched themselves on the back of the van to read/write comfortably, leading to the driver driving off with a man in the back of the van |
| Partially appropriate  **- Either (a) OR (b) need to be fulfilled** | 1 | **(a) Description of both Harold’s (sits in the van) and the driver’s (drives away) behaviour**  **(b) Recognition that the driver did not know (e.g., pay attention, realise) that Harold was in the van. This can be implicit via stressing that the driver was deaf in relation to him driving away**   - He needed somewhere to write so he got into the truck and the truck driver drove away |
| Inappropriate  **- Neither (a) OR (b) need to be fulfilled** | 0 | **(a) Description of both Harold’s (sits in the van) and the driver’s (drives away) behaviour  (b) Recognition that the driver did not know (e.g., pay attention, realise) that Harold was in the van. This can be implicit via stressing that the driver was deaf in relation to him driving away**   - The chap was delivering some clean towels to don’t know, a hotel or something or other, Harold for some reason had a clipboard at the back marking up towels, counting up towels…but he’s obviously not to do with the towel company or maybe actually he might be the assistant! But then the other guy wouldn’t drive off without his assistant. So yeah something to do with towel delivery really |

**CLIP 3: Mannequin – Deliveryman**

|  | Score | Example |
| --- | --- | --- |
| Appropriate  **-Both (a) and(b) need to be fulfilled** | 2 | **(a) Explicit reference to Harold pretending to be a mannequin in order to a accomplish goal (e.g., change time on the clock, or non-specified reasons). Can be implicit if participant mentions pretending + act of changing clock within the same sentence.**  **(b) Understanding that Harold is being discovered by deliveryman plus mention of deliveryman’s reaction/ emotion (e.g., scared or realises Harold was a real person).**   - Harold was pretending to be a mannequin in order to gain access to that building for unknown reasons, except he sneezes leading to the man who is transporting him to realise he's not a mannequin and panic. |
| Partially appropriate  **- Either (a) OR (b) need to be fulfilled** | 1 | **(a) Explicit reference to Harold pretending to be a mannequin in order to accomplish a goal (e.g., change time on the clock, or non-specified reasons). Can be implicit if participant mentions pretending + act of changing clock within the same sentence.**  **(b) Understanding that Harold is being discovered by deliveryman (+ mention of deliveryman’s reaction/ emotion (e.g., scared or realises Harold was a real person).**   - Not quite sure. Was he putting the clock back to punch into his work or something? I couldn’t quite follow it. And so he pretended to be a model to get into the building |
| Inappropriate  **- Neither (a) OR (b) need to be fulfilled** | 0 | **(a) Explicit reference to Harold pretending to be a mannequin in order to accomplish a goal (e.g., change time on the clock, or non-specified reasons). Can be implicit if participant mentions pretending + act of changing clock within the same sentence.**  **(b) Understanding that Harold is being discovered by deliveryman (+ mention of deliveryman’s reaction/ emotion (e.g., scared or realises Harold was a real person).**   - I think it was a mannequin he was coming in with and then I don’t know what really happened |

**CLIP 4: Harold Cat**

| Category | Score | Example |
| --- | --- | --- |
| Appropriate  **-Noth (a) and(b) need to be fulfilled** | 2 | **(a) Recognition that Harold assumes the cat/ fur is the woman’s scarf (e.g., picks it up by mistake, doesn’t know etc)**  **(b) Description of Harold’s / Woman’s reaction when finding out it was a cat**   - In a crowd shop, the salesman is talking to a woman. The woman's cape suddenly drops on the flour. The salesman helps to take the cape. However, he sees the cat's tail as the cape and grab the cat to the woman and makes her a big shock. The woman put the cat down and then take the cape by herself. |
| Partially appropriate  **-Either (a) or (b) need to be fulfilled** | 1 | **(a)Recognition that Harold assumes the cat/ fur is the woman’s scarf (e.g., picks it up by mistake, doesn’t know etc)**  **(b)Description of Harold’s / Woman’s reaction when finding out it was a cat**   - Harold accidentally knocked the woman's scarf off which fell on the floor. He attempted to pick it up but unknowingly handed the woman a similar looking cat instead. She dropped the cat and put on her scarf. |
| Inappropriate  **-Neither (a) or (b) need to be fulfilled** | 0 | **(a) Recognition that Harold assumes the cat/ fur is the woman’s scarf (e.g., picks it up by mistake, doesn’t know etc)**  **(b) Description of Harold’s / Woman’s reaction when finding out it was a cat**   - maybe they are clothes lying there, or maybe skin - animal skin - A woman got handed a cat to wear - There was a sale and the man handed her a cat. |

**CLIP 5: Fan Mildred**

| Category | Score | Example |
| --- | --- | --- |
| Appropriate  **-Both (a) and (b) need to be fulfilled** | 2 | 1. **Harold’s & / Mildred’s actions (e.g., pretending to faint) in *relation* to the other people coming in** 2. **Either the reaction of the incoming people (e.g., concerned)  OR insight into what the men coming in would mean (e.g., trouble).**  - A man and a women were talking when another man walks in. Harold becomes scared/ shocked and tells the lady to close her eyes and sits down. He then starts fanning her and the other man looks shocked and worried about the woman. Harold then tells the man to be quiet |
| **Partially appropriate**  **-Either (a) or (b) need to be fulfilled** | 1 | 1. **Harold’s & / Mildred’s actions (e.g., pretending to faint) in *relation* to the other people coming in** 2. **Either the reaction of the incoming people (e.g., concerned)  OR insight into what the men coming in would mean (e.g., trouble).**  - It looked like he was having some sort of rendezvous with her and she wasn’t meant to be there so they’re pretending that she has passed out |
| **Inappropriate**  **- Neither (a), (b) or need to be fulfilled** | 0 | 1. **Harold’s & / Mildred’s actions (e.g., pretending to faint) in *relation* to the other people coming in** 2. **Either the reaction of the incoming people (e.g., concerned)  OR insight into what the men coming in would mean (e.g., trouble).**  - I’m not quite sure, actually! It looks like … I’m not sure if she actually fainted or pretended to faint in order to attract attention - The man was not supposed to be there with the lady so he had to pretend that he was helping her as she was not feeling well. |

1. **Original SFT Coding Scheme (for clip-specific questions)**

**Silent Film Task (Devine & Hughes, 2013)**

**General Guidance**

| Category | Score | Rule |
| --- | --- | --- |
| Uninterpretable | -99 | - If the candidate does not provide sufficient information in their answer, rate the answer as ‘uninterpretable’ or missing. |
| Fail | 0 | - Answers that miss the point or are factually incorrect. Answers that describe the actions of the clip without evidence of mentalizing. - Inappropriate mentalizing (i.e., answers that contain an explicit mental state but is wrong with respect to the clip). This includes reality errors. - Over-interpretation of the clip (e.g., attribution of intentions/mental states not evident in the content of the clip). |
| Partial | 1 | - Answers that are technically correct but do not demonstrate full understanding of mental states (e.g., answers that imply mental states but do not explicitly use mental-state terms). - Answers that refer to motivations or low-level mental states (e.g., desires rather than cognitions). |
| Pass | 2 | - An explicit mental-state attribution, appropriate to the context of the clip. |

If there is more than one answer, assign the higher score. Do not penalize for poor spelling or grammar.

1. Why did the men hide?

|  | | Examples |
| --- | --- | --- |
| Fail | 0 | **Description/No Mentalizing:** The woman is after them. They’re not meant to be in there. The woman was coming. They did something wrong. They are doing something suspicious. They owe something. They cannot pay. They need to pay. To hide from the woman (repeats question).  **Inappropriate mentalizing/over-interpretation**: The men were frightened of the woman; They didn’t like the lady; They might be keeping a secret; They didn’t want anybody else in the room; They were stealing or spying; They’ve sneaked into the room. The woman will scold them. |
| Partial | 1 | **Implicit mentalizing:** They are avoiding the woman for some reason. They do not want to pay. The woman is mad/angry at them.  **Under-mentalizing:** The men didn’t want to get caught. The men didn’t want to pay the woman. The men didn’t want the woman to see them. They didn’t want the woman to see them. |
| Pass | 2 | **Explicit mentalizing:** They wanted the woman to think/believe they were not in/at home. They didn’t want the woman to know they were home/what they were doing. They didn’t want the woman to find them (find/found are considered cognitive terms). So they do not get found by the woman. |

1. What does the woman think?

|  | | Examples |
| --- | --- | --- |
| Fail | 0 | **Description/No Mentalizing:** She is looking for something. She is thinking (repeats question). She heard a noise. What are they doing? Terrible men!  **Inappropriate mentalizing/over-interpretation**: There is someone in the house. She thinks they hid. She thinks they ran away. Who is in here? She thinks there are thieves in the room. She is worried. She is going to punish them for not giving money. The men stole something. |
| Partial | 1 | **Implicit mentalizing:** She thinks she is going crazy. She thought they were at home (facts about what she thought before coming in). She thought she heard something (facts about before she entered the room). She is confused. “What’s going on?” (confusion).  **Under-mentalizing:** She wants to see the men. She is angry (emotion). She wants to tell them off. She is surprised. |
| Pass | 2 | **Explicit mentalizing:** She thinks they are not at home.  **Adopts voice of woman:** “Where are they?”; “They’re not here”. “Why is there no one here?” |

1. Why did the driver lock Harold in the van?

|  | | Examples |
| --- | --- | --- |
| Fail | 0 | **Description/No Mentalizing:** The man is deaf/hard of hearing. He was reading on the van. He wasn’t supposed to be there. The towels will fall out. He was in a rush.  **Inappropriate mentalizing/over-interpretation**: The man told him to. He kidnapped him. He wanted to punish Harold. |
| Partial | 1 | **Implicit mentalizing:** He did not see/hear him.  **Under-mentalizing:** He wanted to/had to continue on his rounds. He doesn’t want the towels to fall out. |
| Pass | 2 | **Explicit mentalizing:** The driver didn’t know Harold was in the van. He didn’t mean/intend to. He did not notice Harold was in the van. It was an accident. He wasn’t paying attention. He was careless |

1. What is the deliveryman feeling and why?

|  | | Examples |
| --- | --- | --- |
| Fail | 0 | **Description/No Mentalizing:** He is dressed in women’s clothes. The model moved. He is tired from carrying Harold.  **Inappropriate mentalizing/over-interpretation**: He is bored as he always does the same work. He is angry because he fought with Harold. ~~He didn’t know he was there~~. He has no idea. The delivery man is angry because he was tricked (suggests he knows he was tricked). |
| Partial | 1 | **Under-mentalizing:** Identifies correct emotion OR references deliveryman’s lack of knowledge/being deceived. Scared because it was alive (no reference to belief/thought). Shocked because he thought it was a woman (incorrect belief). Scared because he never saw a mannequin moving (no reference to belief/thought). Shocked because it didn’t look like a real man (correct emotion, no explicit mention of belief). No emotion but adopts voice of man (“Why is it moving!”). |
| Pass | 2 | **Explicit mentalizing:** Correct emotion (scared/shocked/frightened/surprised/confused/anxious/worried) and reference to deliveryman’s lack of knowledge/being deceived (e.g., he thought it was a mannequin/dummy but it was a man; he thought it came to life, he didn’t know the man was there). |

1. Why did Harold pick up the cat?

|  | | Examples |
| --- | --- | --- |
| Fail | 0 | **Description/No Mentalizing:** The lady dropped her cat. Cats are not allowed in the shop. He was rushing. He picked up the cat instead of the scarf.  **Inappropriate mentalizing/over-interpretation**: He wanted to give her back her cat. He wanted to scare/trick/distract her. He thought it was a cat. He wanted someone to buy it. He wanted to keep the scarf for himself. He hates cats. He did it on purpose. |
| Partial | 1 | **Implicit mentalizing:** It was beside her scarf. It looked like the scarf. It was the same colour.  **Under-mentalizing:** He wanted to give her back her scarf. He didn’t understand. |
| Pass | 2 | **Explicit mentalizing:** He thought it was the woman’s scarf. He didn’t realise/know it was a cat. He was confused. He mistakenly picked up the cat. He thought it was a scarf. |

1. Why does Harold fan Mildred?

|  | | Examples |
| --- | --- | --- |
| Fail | 0 | **Description/No Mentalizing:** Mildred was tired/hot. The room was too hot. Mildred fainted. To cool Mildred down. He worked for Mildred/It was his job. The man is coming.  **Inappropriate mentalizing/over-interpretation**: To impress the man. Mildred wanted to rest. Mildred is pretending to faint/be hot. The boss/man does not want him to be with Mildred. Harold is trying to get attention. Harold wasn’t working so Mildred pretended to be sick. Mildred didn’t want the man to know they were talking. |
| Partial | 1 | **Implicit mentalizing:** Harold was surprised/shocked to see the man come in. To hide that he likes/fancies Mildred. He loves/likes her. To make it look like she has fainted/he is looking after her (implies attempt to alter man’s perception).  **Under-mentalizing:** He doesn’t want his boss to catch him/get in trouble. He doesn’t want the man to see them together. Harold doesn’t want to get fired. |
| Pass | 2 | **Explicit mentalizing:** Harold wants the man to think Mildred fainted. Harold is pretending/acting she fainted/is hot/asleep. Harold is trying to trick the man. So that the man doesn’t know what they are doing/that they are together. To trick the man coming in. They didn’t want the man to notice. |
